# Supplementary figures and images for: Augmenting complex and dynamic performance through mindfulness-based cognitive training: An evaluation of training adherence, trait mindfulness, personality and resting-state EEG
Source: PLoS One. 2024 May 20;19(5):e0292501. doi: 10.1371/journal.pone.0292501 (PMC11104625; doi:10.1371/journal.pone.0292501)

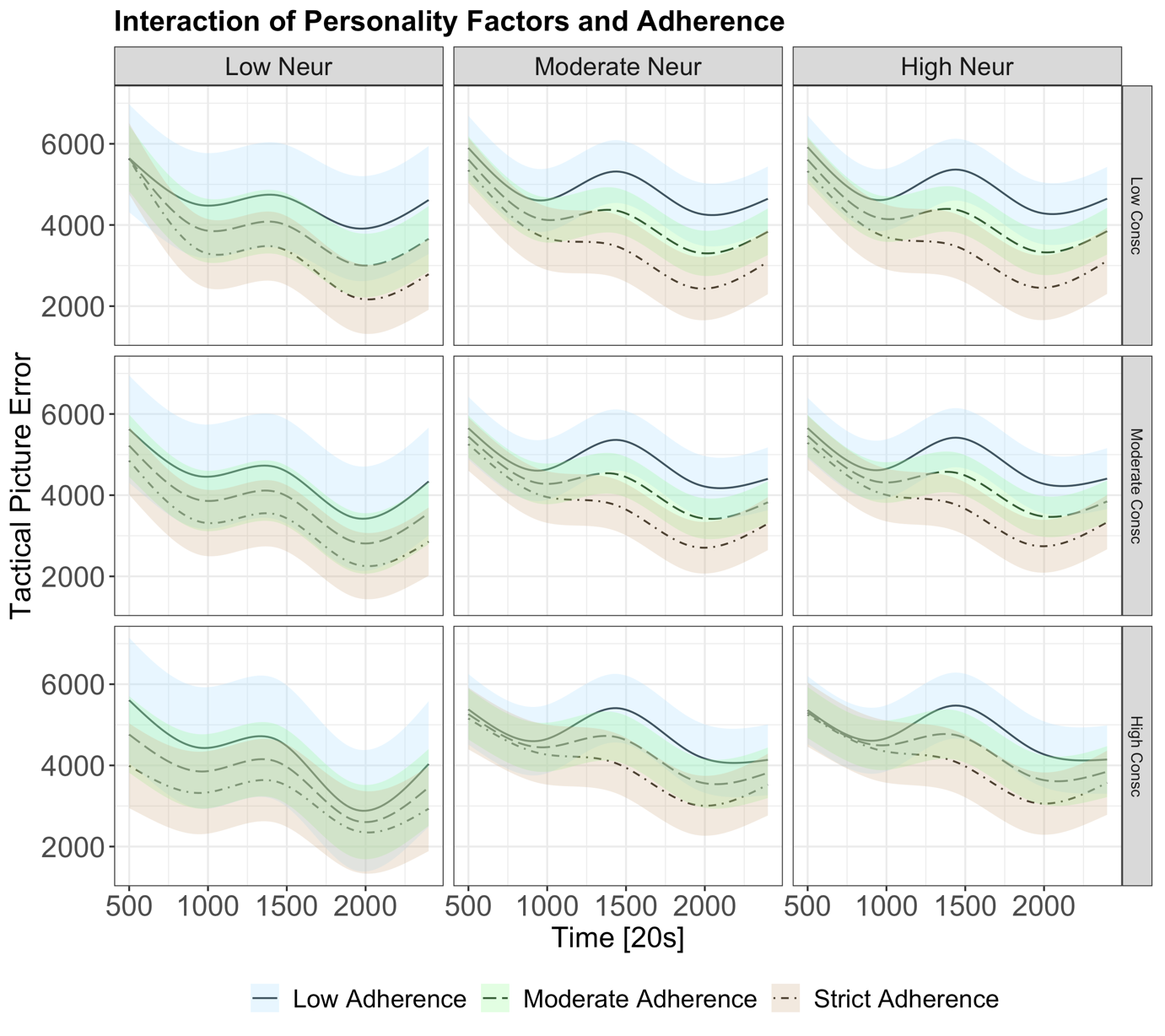

Supplement: S1 Fig — Modelled effects of conscientiousness and neuroticism on TPE scores. TPE is represented on the y-axis (higher scores indicate poorer performance), while experimental time is represented on the x-axis. Colours indicate varying levels of adherence in the cognitive training intervention (brown indicates high levels of adherence, green for moderate and blue for low adherence). Facets represent varying levels of neuroticism (left to right = low to high) and conscientiousness (top to bottom = low to high). Differences in factors are categorised as low, moderate and high based on the sample’s first quartile, median and third quartile, respectively. Statistical models included these predictors as continuous variables, however, categories were created for visualisation purposes. (PNG) [file pone.0292501.s001.png]
