# Supplementary material for: Augmenting complex and dynamic performance through mindfulness-based cognitive training: An evaluation of training adherence, trait mindfulness, personality and resting-state EEG
Source: PLoS One. 2024 May 20;19(5):e0292501. doi: 10.1371/journal.pone.0292501 (PMC11104625; doi:10.1371/journal.pone.0292501)
Supplement: S2 File — (DOCX) [file pone.0292501.s003.docx]

**S2: Regression Model Outputs**

**Table S2.1.** Model estimates for regression predicting post-intervention IAF from pre-intervention IAF.

|  | Estimate | SE | *t-*value | *p-*value |
| --- | --- | --- | --- | --- |
| Intercept | 2.44 | 0.61 | 4.04 | <.001*** |
| Adherence | -0.02 | 0.006 | -2.68 | 0.011* |
| Pre- IAF | 0.77 | 0.061 | 12.71 | <.001*** |

*Note.* * denotes significant p-values (at the 0.05 level), ** at 0.01, *** at 0.001 level.

**Table S2.2.** Model estimates for regression predicting post-intervention 1/*f*  intercept from pre-intervention /*f*  intercept.

|  | Estimate | SE | *t-*value | *p-*value |
| --- | --- | --- | --- | --- |
| Intercept | -6.29 | 2.62 | -2.4 | 0.022* |
| Adherence | 0.014 | 0.012 | 1.157 | 0.255 |
| Pre- 1/*f*  intercept | 0.75 | 0.1 | 7.16 | <.001*** |

*Note.* * denotes significant p-values (at the 0.05 level), ** at 0.01, *** at 0.001 level.

**Table S2.3.** Model estimates for regression predicting post-intervention 1/*f*  slope from pre-intervention 1/*f*  slope.

|  | Estimate | SE | *t-*value | *p-*value |
| --- | --- | --- | --- | --- |
| Intercept | -0.35 | 0.12 | -2.7 | 0.012* |
| Adherence | -0.003 | 0.005 | -0.63 | 0.53 |
| Pre- 1/*f*  slope | 0.65 | 0.1 | 6.42 | <0.001*** |

*Note.* * denotes significant p-values (at the 0.05 level), ** at 0.01, *** at 0.001 level.
