# Supplementary material for: Augmenting complex and dynamic performance through mindfulness-based cognitive training: An evaluation of training adherence, trait mindfulness, personality and resting-state EEG
Source: PLoS One. 2024 May 20;19(5):e0292501. doi: 10.1371/journal.pone.0292501 (PMC11104625; doi:10.1371/journal.pone.0292501)
Supplement: S3 File — (DOCX) [file pone.0292501.s004.docx]

**S4: Linear Mixed Effect Model Outputs**

Table 1: Summary of effects of Adherence on TPE Scores (Type II Wald Tests)

|  | Chisq | Df | Pr(*>*Chisq) |
| --- | --- | --- | --- |
| ns(Time, 4) | 295.756866 | 4 | 0.0000000 |
| training | 5.803218 | 1 | 0.0159969 |
| baseline | 75.857091 | 1 | 0.0000000 |
| decline | 5.266405 | 1 | 0.0217409 |
| ns(Time, 4):training | 121.328248 | 4 | 0.0000000 |

Table 2: Parameters from model produced by the call lmer(formula = TPE ~ ns(Time, 4) * training + baseline + decline + (1 | subj), data = baselined)

|  | Estimate | Std. Error | df | t value | Pr(*>\|*t*\|*) | sd subj |
| --- | --- | --- | --- | --- | --- | --- |
| (Intercept) | 6.9e+03 | 8.7e+02 | 44 | 7.9 | 6e-10 | 2e+03 |
| ns(Time, 4)1 | 9.2e+02 | 2.4e+02 | 3.8e+03 | 3.8 | 0.00015 |  |
| ns(Time, 4)2 | -2.3e+02 | 2.4e+02 | 3.8e+03 | -0.95 | 0.34 |  |
| ns(Time, 4)3 | -1.7e+03 | 4.9e+02 | 3.8e+03 | -3.4 | 0.00074 |  |
| ns(Time, 4)4 | -4.8e+02 | 2.3e+02 | 3.8e+03 | -2.1 | 0.037 |  |
| training | -31 | 51 | 44 | -0.61 | 0.55 |  |
| baseline | 0.12 | 0.014 | 3.8e+03 | 8.7 | 4.5e-18 |  |
| decline | 78 | 34 | 37 | 2.3 | 0.027 |  |
| ns(Time, 4)1:training | -1.2e+02 | 20 | 3.8e+03 | -6.1 | 1e-09 |  |
| ns(Time, 4)2:training | -1.5e+02 | 19 | 3.8e+03 | -7.6 | 4.6e-14 |  |
| ns(Time, 4)3:training | -1.3e+02 | 39 | 3.8e+03 | -3.5 | 0.00056 |  |
| ns(Time, 4)4:training | -30 | 19 | 3.8e+03 | -1.6 | 0.11 |  |

Table 3: Summary of effects of Adherence and Skew on TPE Scores (Type II Wald Tests)

|  | Chisq | Df | Pr(¿Chisq) |
| --- | --- | --- | --- |
| ns(Time, 4) | 303.4828710 | 4 | 0.0000000 |
| training | 1.8247145 | 1 | 0.1767531 |
| skew | 0.0025903 | 1 | 0.9594095 |
| baseline | 49.6591057 | 1 | 0.0000000 |
| decline | 4.6577640 | 1 | 0.0309134 |
| ns(Time, 4):training | 27.7002483 | 4 | 0.0000143 |
| ns(Time, 4):skew | 44.7575312 | 4 | 0.0000000 |
| training:skew | 0.7070632 | 1 | 0.4004205 |
| ns(Time, 4):training:skew | 25.2481455 | 4 | 0.0000448 |

Table 4: Parameters from model produced by the call lmer(formula = TPE ~ ns(Time, 4) * training * skew + baseline + decline + (1 | subj), data = data with skew)

|  | Estimate | Std. Error | df | t value | Pr(*>\|*t*\|*) | sd subj |
| --- | --- | --- | --- | --- | --- | --- |
| (Intercept) | 1e+04 | 1.8e+03 | 37 | 5.6 | 2.2e-06 | 1.9e+03 |
| ns(Time, 4)1 | -9.6e+02 | 6.2e+02 | 3.4e+03 | -1.6 | 0.12 |  |
| ns(Time, 4)2 | -1.2e+03 | 6.2e+02 | 3.4e+03 | -2 | 0.049 |  |
| ns(Time, 4)3 | -7.4e+03 | 1.2e+03 | 3.4e+03 | -6 | 2.9e-09 |  |
| ns(Time, 4)4 | -3.8e+02 | 5.8e+02 | 3.4e+03 | -0.65 | 0.52 |  |
| training | -2.4e+02 | 1.1e+02 | 38 | -2.2 | 0.034 |  |
| skew | -2.1e+03 | 1.1e+03 | 38 | -1.9 | 0.064 |  |
| baseline | 0.11 | 0.016 | 3.4e+03 | 7 | 2.2e-12 |  |
| decline | 78 | 36 | 31 | 2.2 | 0.039 |  |
| ns(Time, 4)1:training | 36 | 45 | 3.4e+03 | 0.81 | 0.42 |  |
| ns(Time, 4)2:training | -87 | 45 | 3.4e+03 | -2 | 0.05 |  |
| ns(Time, 4)3:training | 2.8e+02 | 90 | 3.4e+03 | 3.1 | 0.0021 |  |
| ns(Time, 4)4:training | -2.8 | 43 | 3.4e+03 | -0.066 | 0.95 |  |
| ns(Time, 4)1:skew | 6.6e+02 | 4.5e+02 | 3.4e+03 | 1.5 | 0.14 |  |
| ns(Time, 4)2:skew | 4.8e+02 | 4.5e+02 | 3.4e+03 | 1.1 | 0.28 |  |
| ns(Time, 4)3:skew | 4e+03 | 9e+02 | 3.4e+03 | 4.4 | 1.1e-05 |  |
| ns(Time, 4)4:skew | -3.4e+02 | 4.3e+02 | 3.4e+03 | -0.81 | 0.42 |  |
| training:skew | 70 | 64 | 38 | 1.1 | 0.28 |  |
| ns(Time, 4)1:training:skew | 43 | 26 | 3.4e+03 | 1.6 | 0.1 |  |
| ns(Time, 4)2:training:skew | -10 | 26 | 3.4e+03 | -0.4 | 0.69 |  |
| ns(Time, 4)3:training:skew | -82 | 53 | 3.4e+03 | -1.6 | 0.12 |  |
| ns(Time, 4)4:training:skew | 97 | 25 | 3.4e+03 | 3.9 | 0.0001 |  |

Table 5: Summary of effects of Adherence and Sleep on TPE Scores (Type II Wald Tests)

|  | Chisq | Df | Pr(*>*Chisq) |
| --- | --- | --- | --- |
| ns(Time, 4) | 304.4185480 | 4 | 0.0000000 |
| training | 5.0006690 | 1 | 0.0253375 |
| prior | 0.0980910 | 1 | 0.7541327 |
| baseline | 71.5100810 | 1 | 0.0000000 |
| decline | 4.8522084 | 1 | 0.0276108 |
| ns(Time, 4):training | 43.4132725 | 4 | 0.0000000 |
| ns(Time, 4):prior | 79.5273259 | 4 | 0.0000000 |
| training:prior | 0.2797363 | 1 | 0.5968741 |
| ns(Time, 4):training:prior | 36.5678764 | 4 | 0.0000002 |

Table 6: Parameters from model produced by the call lmer(formula = TPE ~ ns(Time, 4) * training * prior + baseline + decline + (1 | subj), data = baselined sleep)

|  | Estimate | Std. Error | df | t value | Pr(*>\|*t*\|*) | sd subj |
| --- | --- | --- | --- | --- | --- | --- |
| (Intercept) | 7.2e+03 | 9.1e+02 | 41 | 8 | 7.1e-10 | 2e+03 |
| ns(Time, 4)1 | 4.3e+02 | 2.5e+02 | 3.8e+03 | 1.7 | 0.092 |  |
| ns(Time, 4)2 | -6e+02 | 2.5e+02 | 3.8e+03 | -2.4 | 0.018 |  |
| ns(Time, 4)3 | -2e+03 | 5.1e+02 | 3.8e+03 | -3.9 | 9.7e-05 |  |
| ns(Time, 4)4 | -8.7e+02 | 2.4e+02 | 3.8e+03 | -3.6 | 0.00029 |  |
| training | -45 | 60 | 41 | -0.75 | 0.45 |  |
| prior[0] | -1.2e+03 | 6.5e+02 | 42 | -1.8 | 0.08 |  |
| baseline | 0.12 | 0.014 | 3.8e+03 | 8.5 | 3.9e-17 |  |
| decline | 77 | 35 | 35 | 2.2 | 0.034 |  |
| ns(Time, 4)1:training | -89 | 22 | 3.8e+03 | -4 | 6e-05 |  |
| ns(Time, 4)2:training | -1.3e+02 | 22 | 3.8e+03 | -5.9 | 3e-09 |  |
| ns(Time, 4)3:training | -1.1e+02 | 44 | 3.8e+03 | -2.6 | 0.0098 |  |
| ns(Time, 4)4:training | 19 | 21 | 3.8e+03 | 0.87 | 0.38 |  |
| ns(Time, 4)1:prior[0] | 1.5e+03 | 2.5e+02 | 3.8e+03 | 6 | 2.9e-09 |  |
| ns(Time, 4)2:prior[0] | 1.3e+03 | 2.5e+02 | 3.8e+03 | 5.2 | 2.7e-07 |  |
| ns(Time, 4)3:prior[0] | 9.3e+02 | 5e+02 | 3.8e+03 | 1.8 | 0.066 |  |
| ns(Time, 4)4:prior[0] | 5.8e+02 | 2.4e+02 | 3.8e+03 | 2.4 | 0.016 |  |
| training:prior[0] | 74 | 57 | 42 | 1.3 | 0.2 |  |
| ns(Time, 4)1:training:prior[0] | -81 | 22 | 3.8e+03 | -3.6 | 0.00028 |  |
| ns(Time, 4)2:training:prior[0] | -81 | 22 | 3.8e+03 | -3.7 | 0.00025 |  |
| ns(Time, 4)3:training:prior[0] | -49 | 44 | 3.8e+03 | -1.1 | 0.27 |  |
| ns(Time, 4)4:training:prior[0] | 12 | 21 | 3.8e+03 | 0.54 | 0.59 |  |

Table 7: Summary of effects of Adherence and Enjoyment on TPE Scores (Type II Wald Tests)

|  | Chisq | Df | Pr(*>*Chisq) |
| --- | --- | --- | --- |
| ns(Time, 4) | 226.8284966 | 4 | 0.0000000 |
| training | 5.3512045 | 1 | 0.0207080 |
| enjoy | 0.6540723 | 1 | 0.4186605 |
| baseline | 110.4019750 | 1 | 0.0000000 |
| decline | 4.6561632 | 1 | 0.0309423 |
| ns(Time, 4):training | 144.3474762 | 4 | 0.0000000 |
| ns(Time, 4):enjoy | 37.7048978 | 4 | 0.0000001 |
| training:enjoy | 2.2120540 | 1 | 0.1369362 |
| ns(Time, 4):training:enjoy | 80.5939871 | 4 | 0.0000000 |

Table 8: Parameters from model produced by the call lmer(formula = TPE ~ ns(Time, 4) * training * enjoy + baseline + decline + (1 | subj), data = baselined enjoy)

|  | Estimate | Std. Error | df | t value | Pr(*>\|*t*\|*) | sd subj |
| --- | --- | --- | --- | --- | --- | --- |
| (Intercept) | -1.6e+03 | 2.9e+03 | 42 | -0.57 | 0.57 | 1.8e+03 |
| ns(Time, 4)1 | 7.6e+03 | 1.2e+03 | 3.6e+03 | 6.1 | 9.1e-10 |  |
| ns(Time, 4)2 | -5.1e+02 | 1.2e+03 | 3.6e+03 | -0.42 | 0.68 |  |
| ns(Time, 4)3 | 5.3e+03 | 2.5e+03 | 3.6e+03 | 2.1 | 0.034 |  |
| ns(Time, 4)4 | 8.3e+03 | 1.1e+03 | 3.6e+03 | 7.3 | 5.1e-13 |  |
| training | 6.5e+02 | 2.1e+02 | 40 | 3.1 | 0.0039 |  |
| enjoy | 1.2e+02 | 44 | 41 | 2.7 | 0.011 |  |
| baseline | 0.16 | 0.015 | 3.6e+03 | 11 | 1.9e-25 |  |
| decline | 71 | 33 | 33 | 2.2 | 0.038 |  |
| ns(Time, 4)1:training | -7.5e+02 | 86 | 3.6e+03 | -8.7 | 6e-18 |  |
| ns(Time, 4)2:training | -2.5e+02 | 86 | 3.6e+03 | -2.9 | 0.0034 |  |
| ns(Time, 4)3:training | -9.5e+02 | 1.7e+02 | 3.6e+03 | -5.5 | 3.7e-08 |  |
| ns(Time, 4)4:training | -5.6e+02 | 81 | 3.6e+03 | -6.9 | 5.9e-12 |  |
| ns(Time, 4)1:enjoy | -1e+02 | 18 | 3.6e+03 | -5.6 | 2.5e-08 |  |
| ns(Time, 4)2:enjoy | 11 | 18 | 3.6e+03 | 0.63 | 0.53 |  |
| ns(Time, 4)3:enjoy | -99 | 37 | 3.6e+03 | -2.7 | 0.0073 |  |
| ns(Time, 4)4:enjoy | -1.3e+02 | 17 | 3.6e+03 | -7.5 | 5.6e-14 |  |
| training:enjoy | -9.7 | 3 | 41 | -3.2 | 0.0026 |  |
| ns(Time, 4)1:training:enjoy | 9.5 | 1.3 | 3.6e+03 | 7.5 | 7.8e-14 |  |
| ns(Time, 4)2:training:enjoy | 1.1 | 1.2 | 3.6e+03 | 0.87 | 0.39 |  |
| ns(Time, 4)3:training:enjoy | 12 | 2.5 | 3.6e+03 | 4.6 | 3.9e-06 |  |
| ns(Time, 4)4:training:enjoy | 7.8 | 1.2 | 3.6e+03 | 6.6 | 3.6e-11 |  |

Table 9: Summary of effects of Adherence and MAAS on TPE Scores (Type II Wald Tests)

|  | Chisq | Df | Pr(*>*Chisq) |
| --- | --- | --- | --- |
| ns(Time, 4) | 356.875012 | 4 | 0.0000000 |
| training | 4.621087 | 1 | 0.0315812 |
| MAAS | 1.416625 | 1 | 0.2339597 |
| decline | 2.496654 | 1 | 0.1140885 |
| baseline | 56.894530 | 1 | 0.0000000 |
| ns(Time, 4):training | 95.784333 | 4 | 0.0000000 |
| ns(Time, 4):MAAS | 25.038805 | 4 | 0.0000494 |
| training:MAAS | 0.967150 | 1 | 0.3253920 |
| ns(Time, 4):training:MAAS | 99.063252 | 4 | 0.0000000 |

Table 10: Parameters from model produced by the call lmer(formula = TPE ~ ns(Time, 4) * training * MAAS + decline

+ baseline + (1 | subj), data = baselined)

|  | Estimate | Std. Error | df | t value | Pr(*>\|*t*\|*) | sd subj |
| --- | --- | --- | --- | --- | --- | --- |
| (Intercept) | 1.3e+04 | 3e+03 | 40 | 4.2 | 0.00014 | 2e+03 |
| ns(Time, 4)1 | -2.5e+03 | 1.2e+03 | 3.6e+03 | -2.1 | 0.034 |  |
| ns(Time, 4)2 | -3.9e+03 | 1.2e+03 | 3.6e+03 | -3.3 | 0.001 |  |
| ns(Time, 4)3 | -4.2e+03 | 2.4e+03 | 3.6e+03 | -1.8 | 0.08 |  |
| ns(Time, 4)4 | -4.5e+03 | 1.1e+03 | 3.6e+03 | -4 | 7.8e-05 |  |
| training | -3.9e+02 | 2.3e+02 | 39 | -1.7 | 0.1 |  |
| MAAS | -1.8e+03 | 8.9e+02 | 39 | -2 | 0.056 |  |
| decline | 61 | 38 | 33 | 1.6 | 0.12 |  |
| baseline | 0.11 | 0.014 | 3.6e+03 | 7.5 | 5.8e-14 |  |
| ns(Time, 4)1:training | 2.7e+02 | 88 | 3.6e+03 | 3.1 | 0.0021 |  |
| ns(Time, 4)2:training | 1.8e+02 | 87 | 3.6e+03 | 2 | 0.041 |  |
| ns(Time, 4)3:training | -30 | 1.8e+02 | 3.6e+03 | -0.17 | 0.86 |  |
| ns(Time, 4)4:training | 4.6e+02 | 83 | 3.6e+03 | 5.6 | 2.4e-08 |  |
| ns(Time, 4)1:MAAS | 1e+03 | 3.5e+02 | 3.6e+03 | 3 | 0.0032 |  |
| ns(Time, 4)2:MAAS | 1e+03 | 3.4e+02 | 3.6e+03 | 3 | 0.003 |  |
| ns(Time, 4)3:MAAS | 6.7e+02 | 6.9e+02 | 3.6e+03 | 0.97 | 0.33 |  |
| ns(Time, 4)4:MAAS | 1e+03 | 3.3e+02 | 3.6e+03 | 3.2 | 0.0015 |  |
| training:MAAS | 99 | 62 | 39 | 1.6 | 0.12 |  |
| ns(Time, 4)1:training:MAAS | -1.1e+02 | 24 | 3.6e+03 | -4.5 | 6.5e-06 |  |
| ns(Time, 4)2:training:MAAS | -90 | 24 | 3.6e+03 | -3.8 | 0.00017 |  |
| ns(Time, 4)3:training:MAAS | -28 | 48 | 3.6e+03 | -0.58 | 0.56 |  |
| ns(Time, 4)4:training:MAAS | -1.3e+02 | 23 | 3.6e+03 | -5.8 | 8e-09 |  |

Table 11: Summary of effects of Adherence, Neuroticism and Conscientious- ness on TPE Scores (Type II Wald Tests)

|  | Chisq | Df | Pr(*>*Chisq) |
| --- | --- | --- | --- |
| ns(Time, 4) | 291.4279041 | 4 | 0.0000000 |
| training | 2.6392607 | 1 | 0.1042520 |
| neuroticism | 0.9900432 | 1 | 0.3197318 |
| conscientiousness | 0.0111115 | 1 | 0.9160497 |
| baseline | 65.1465269 | 1 | 0.0000000 |
| decline | 1.8878627 | 1 | 0.1694432 |
| ns(Time, 4):training | 171.3433248 | 4 | 0.0000000 |
| ns(Time, 4):neuroticism | 19.2829846 | 4 | 0.0006914 |
| training:neuroticism | 0.0066960 | 1 | 0.9347824 |
| ns(Time, 4):conscientiousness | 75.8102299 | 4 | 0.0000000 |
| training:conscientiousness | 0.7305546 | 1 | 0.3927035 |
| neuroticism:conscientiousness | 0.6388340 | 1 | 0.4241333 |
| ns(Time, 4):training:neuroticism | 42.9025861 | 4 | 0.0000000 |
| ns(Time, 4):training:conscientiousness | 33.2611536 | 4 | 0.0000011 |
| ns(Time, 4):neuroticism:conscientiousness | 15.5681513 | 4 | 0.0036569 |
| training:neuroticism:conscientiousness | 0.1168597 | 1 | 0.7324657 |
| ns(Time, 4):training:neuroticism:conscientiousness | 131.7264513 | 4 | 0.0000000 |

Table 12: Parameters from model produced by the call lmer(formula = TPE ~ ns(Time, 4) * training * neuroticism * conscientiousness + baseline + decline + (1 | subj), data = baselined person)

|  | Estimate | Std. Error | df | t value | Pr(*>\|*t*\|*) | sd subj |
| --- | --- | --- | --- | --- | --- | --- |
| (Intercept) | -2e+04 | 2.2e+04 | 35 | -0.89 | 0.38 | 2.1e+03 |
| ns(Time, 4)1 | 1.4e+04 | 7.8e+03 | 3.7e+03 | 1.7 | 0.085 |  |
| ns(Time, 4)2 | 5.7e+04 | 7.8e+03 | 3.7e+03 | 7.4 | 1.6e-13 |  |
| ns(Time, 4)3 | 6.9e+04 | 1.6e+04 | 3.7e+03 | 4.4 | 1.3e-05 |  |
| ns(Time, 4)4 | 1.2e+04 | 7.3e+03 | 3.7e+03 | 1.6 | 0.11 |  |
| training | 3.9e+03 | 1.6e+03 | 35 | 2.4 | 0.022 |  |
| neuroticism | 1.5e+03 | 1.1e+03 | 35 | 1.3 | 0.19 |  |
| conscientiousness | 8.9e+02 | 7.3e+02 | 35 | 1.2 | 0.23 |  |
| baseline | 0.11 | 0.014 | 3.7e+03 | 8.1 | 9.3e-16 |  |
| decline | 57 | 42 | 30 | 1.4 | 0.18 |  |
| ns(Time, 4)1:training | -2.7e+03 | 5.8e+02 | 3.7e+03 | -4.6 | 4.1e-06 |  |
| ns(Time, 4)2:training | -5.1e+03 | 5.7e+02 | 3.7e+03 | -9 | 4.7e-19 |  |
| ns(Time, 4)3:training | -8.5e+03 | 1.2e+03 | 3.7e+03 | -7.3 | 3e-13 |  |
| ns(Time, 4)4:training | -1.7e+03 | 5.4e+02 | 3.7e+03 | -3.1 | 0.0017 |  |
| ns(Time, 4)1:neuroticism | -7.9e+02 | 4e+02 | 3.7e+03 | -2 | 0.048 |  |
| ns(Time, 4)2:neuroticism | -3e+03 | 3.9e+02 | 3.7e+03 | -7.8 | 1e-14 |  |
| ns(Time, 4)3:neuroticism | -3.7e+03 | 8e+02 | 3.7e+03 | -4.7 | 2.9e-06 |  |
| ns(Time, 4)4:neuroticism | -4.7e+02 | 3.7e+02 | 3.7e+03 | -1.3 | 0.2 |  |
| training:neuroticism | -2.1e+02 | 87 | 35 | -2.4 | 0.023 |  |
| ns(Time, 4)1:conscientiousness | -6.2e+02 | 2.6e+02 | 3.7e+03 | -2.4 | 0.019 |  |
| ns(Time, 4)2:conscientiousness | -2.2e+03 | 2.6e+02 | 3.7e+03 | -8.5 | 2e-17 |  |
| ns(Time, 4)3:conscientiousness | -2.4e+03 | 5.2e+02 | 3.7e+03 | -4.7 | 3.1e-06 |  |
| ns(Time, 4)4:conscientiousness | -4.1e+02 | 2.4e+02 | 3.7e+03 | -1.7 | 0.09 |  |
| training:conscientiousness | -1.4e+02 | 56 | 35 | -2.5 | 0.019 |  |
| neuroticism:conscientiousness | -51 | 37 | 35 | -1.4 | 0.18 |  |
| ns(Time, 4)1:training:neuroticism | 1.3e+02 | 31 | 3.7e+03 | 4.2 | 3.3e-05 |  |
| ns(Time, 4)2:training:neuroticism | 2.5e+02 | 30 | 3.7e+03 | 8.3 | 1.3e-16 |  |
| ns(Time, 4)3:training:neuroticism | 4.1e+02 | 61 | 3.7e+03 | 6.7 | 2.4e-11 |  |
| ns(Time, 4)4:training:neuroticism | 74 | 28 | 3.7e+03 | 2.6 | 0.0097 |  |
| ns(Time, 4)1:training:conscientiousness | 1e+02 | 20 | 3.7e+03 | 5.2 | 2.7e-07 |  |
| ns(Time, 4)2:training:conscientiousness | 1.8e+02 | 20 | 3.7e+03 | 9.4 | 8.5e-21 |  |
| ns(Time, 4)3:training:conscientiousness | 2.8e+02 | 39 | 3.7e+03 | 7.1 | 1.7e-12 |  |
| ns(Time, 4)4:training:conscientiousness | 59 | 19 | 3.7e+03 | 3.2 | 0.0016 |  |
| ns(Time, 4)1:neuroticism:conscientiousness | 36 | 13 | 3.7e+03 | 2.8 | 0.0057 |  |
| ns(Time, 4)2:neuroticism:conscientiousness | 1.2e+02 | 13 | 3.7e+03 | 8.9 | 9.9e-19 |  |
| ns(Time, 4)3:neuroticism:conscientiousness | 1.3e+02 | 26 | 3.7e+03 | 4.9 | 1.1e-06 |  |
| ns(Time, 4)4:neuroticism:conscientiousness | 17 | 12 | 3.7e+03 | 1.4 | 0.17 |  |
| training:neuroticism:conscientiousness | 7.2 | 3 | 35 | 2.4 | 0.02 |  |
| ns(Time, 4)1:training:neuroticism:conscientiousness | -5.1 | 1 | 3.7e+03 | -4.9 | 8.8e-07 |  |
| ns(Time, 4)2:training:neuroticism:conscientiousness | -9.2 | 1 | 3.7e+03 | -9 | 3.9e-19 |  |
| ns(Time, 4)3:training:neuroticism:conscientiousness | -14 | 2.1 | 3.7e+03 | -6.6 | 5.1e-11 |  |
| ns(Time, 4)4:training:neuroticism:conscientiousness | -2.7 | 0.97 | 3.7e+03 | -2.7 | 0.006 |  |

Table 13: Summary of effects of Adherence and IAF on TPE Scores (Type II Wald Tests)

Chisq Df Pr(*>*Chisq)

ns(Time, 4) 308.5934670 4 0.0000000

in cog 0.1546499 1 0.6941308

training 5.7267703 1 0.0167082

baseline 73.4458902 1 0.0000000

decline 4.6599689 1 0.0308738

ns(Time, 4):in cog 29.7466427 4 0.0000055

ns(Time, 4):training 124.6504034 4 0.0000000

in cog:training 1.0188197 1 0.3127991

ns(Time, 4):in cog:training 137.7421910 4 0.0000000

Table 14: Parameters from model produced by the call lmer(formula = TPE ~ ns(Time, 4) * in cog * training + baseline + decline + (1 | subj), data = baselined)

Estimate Std. Error df t value Pr(*>|*t*|*) sd subj

(Intercept) -2.1e+02 8.8e+03 42 -0.023 0.98 2e+03

ns(Time, 4)1 3.1e+03 3.4e+03 3.8e+03 0.91 0.36

ns(Time, 4)2 2.4e+04 3.4e+03 3.8e+03 7.1 1.8e-12 ns(Time, 4)3 -2.1e+04 6.8e+03 3.8e+03 -3.1 0.002

ns(Time, 4)4 -2e+03 3.2e+03 3.8e+03 -0.62 0.53

in cog 7.2e+02 8.8e+02 42 0.82 0.42

training 7.3e+02 7.7e+02 42 0.95 0.35

baseline 0.12 0.014 3.8e+03 8.6 1.5e-17

decline 75 35 35 2.2 0.038

ns(Time, 4)1:in cog -2.2e+02 3.4e+02 3.8e+03 -0.65 0.52

ns(Time, 4)2:in cog -2.4e+03 3.4e+02 3.8e+03 -7.2 8.7e-13 ns(Time, 4)3:in cog 2e+03 6.9e+02 3.8e+03 2.9 0.0043

ns(Time, 4)4:in cog 1.5e+02 3.2e+02 3.8e+03 0.47 0.64

ns(Time, 4)1:training -4.6e+02 3e+02 3.8e+03 -1.5 0.13

ns(Time, 4)2:training -2.2e+03 3e+02 3.8e+03 -7.4 2e-13 ns(Time, 4)3:training 1e+03 6e+02 3.8e+03 1.7 0.09

ns(Time, 4)4:training -3.7e+02 2.8e+02 3.8e+03 -1.3 0.2

in cog:training -77 78 42 -0.99 0.33

ns(Time, 4)1:in cog:training 35 31 3.8e+03 1.1 0.26

ns(Time, 4)2:in cog:training 2.1e+02 30 3.8e+03 6.9 6e-12 ns(Time, 4)3:in cog:training -1.2e+02 61 3.8e+03 -1.9 0.054

ns(Time, 4)4:in cog:training 34 29 3.8e+03 1.2 0.24

Table 15: Summary of effects of Adherence and 1/f Slope on TPE Scores (Type II Wald Tests)

Chisq Df Pr(*>*Chisq)

ns(Time, 4) 305.2294838 4 0.0000000

in slope 0.2215652 1 0.6378499

training 4.7807954 1 0.0287788

baseline 63.1651232 1 0.0000000

decline 4.9540543 1 0.0260296

ns(Time, 4):in slope 73.9759426 4 0.0000000

ns(Time, 4):training 79.4106303 4 0.0000000

in slope:training 0.0349779 1 0.8516420

ns(Time, 4):in slope:training 50.2625614 4 0.0000000

Table 16: Parameters from model produced by the call lmer(formula = TPE ~ ns(Time, 4) * in slope * training + baseline + decline + (1 | subj), data = baselined)

Estimate Std. Error df t value Pr(*>|*t*|*) sd subj

(Intercept) 8.9e+03 2e+03 41 4.4 8.5e-05 2e+03 ns(Time, 4)1 -3.1e+03 7.1e+02 3.8e+03 -4.4 9.1e-06

ns(Time, 4)2 -4.7e+03 7e+02 3.8e+03 -6.7 2.8e-11 ns(Time, 4)3 -3.9e+03 1.4e+03 3.8e+03 -2.7 0.0063

ns(Time, 4)4 -3e+02 6.7e+02 3.8e+03 -0.45 0.65

in slope 1.7e+03 1.5e+03 42 1.1 0.28

training -2.3e+02 1.9e+02 42 -1.3 0.22

baseline 0.11 0.014 3.8e+03 7.9 2.5e-15

decline 79 36 35 2.2 0.033

ns(Time, 4)1:in slope -3.5e+03 5.8e+02 3.8e+03 -6.1 1.5e-09

ns(Time, 4)2:in slope -3.9e+03 5.8e+02 3.8e+03 -6.7 2.2e-11

ns(Time, 4)3:in slope -1.9e+03 1.2e+03 3.8e+03 -1.6 0.11

ns(Time, 4)4:in slope 1.7e+02 5.5e+02 3.8e+03 0.3 0.76

ns(Time, 4)1:training 1.6e+02 72 3.8e+03 2.3 0.022

ns(Time, 4)2:training 2e+02 72 3.8e+03 2.7 0.0063

ns(Time, 4)3:training 75 1.4e+02 3.8e+03 0.52 0.6

ns(Time, 4)4:training 28 69 3.8e+03 0.41 0.68

in slope:training -1.9e+02 1.7e+02 42 -1.1 0.29

ns(Time, 4)1:in slope:training 2.5e+02 67 3.8e+03 3.7 0.00019

ns(Time, 4)2:in slope:training 3.1e+02 67 3.8e+03 4.6 3.8e-06 ns(Time, 4)3:in slope:training 2e+02 1.4e+02 3.8e+03 1.4 0.15

ns(Time, 4)4:in slope:training 62 64 3.8e+03 0.97 0.33

Table 17: Summary of effects of Adherence and 1/f Intercept on TPE Scores (Type II Wald Tests)

Chisq Df Pr(*>*Chisq)

ns(Time, 4) 303.3113934 4 0.0000000

in intercept 0.0057057 1 0.9397883

training 5.3115750 1 0.0211842

baseline 66.5803629 1 0.0000000

decline 4.9833641 1 0.0255922

ns(Time, 4):in intercept 40.3667204 4 0.0000000

ns(Time, 4):training 105.9425391 4 0.0000000

in intercept:training 0.0049174 1 0.9440949

ns(Time, 4):in intercept:training 60.7083392 4 0.0000000

Table 18: Parameters from model produced by the call lmer(formula = TPE ~ ns(Time, 4) * in intercept * training + baseline + decline + (1 | subj), data = baselined)

Estimate Std. Error df t value Pr(*>|*t*|*) sd subj

(Intercept) -7.9e+03 1.9e+04 42 -0.42 0.67 2e+03

ns(Time, 4)1 4.7e+04 7.2e+03 3.8e+03 6.6 5.2e-11

ns(Time, 4)2 2.6e+04 7.2e+03 3.8e+03 3.6 0.00036

ns(Time, 4)3 -3e+03 1.4e+04 3.8e+03 -0.21 0.83

ns(Time, 4)4 -1.5e+03 7e+03 3.8e+03 -0.21 0.83

in intercept -6e+02 7.5e+02 42 -0.79 0.43

training 7.8e+02 1.7e+03 42 0.47 0.64

baseline 0.12 0.014 3.8e+03 8.2 4.5e-16

decline 80 36 35 2.2 0.032

ns(Time, 4)1:in intercept 1.9e+03 2.9e+02 3.8e+03 6.5 1.2e-10

ns(Time, 4)2:in intercept 1e+03 2.9e+02 3.8e+03 3.6 0.00032

ns(Time, 4)3:in intercept -52 5.8e+02 3.8e+03 -0.09 0.93

ns(Time, 4)4:in intercept -38 2.8e+02 3.8e+03 -0.14 0.89

ns(Time, 4)1:training -2.9e+03 6.4e+02 3.8e+03 -4.5 6.6e-06

ns(Time, 4)2:training -2.3e+03 6.4e+02 3.8e+03 -3.6 0.00033

ns(Time, 4)3:training 8e+02 1.3e+03 3.8e+03 0.62 0.54

ns(Time, 4)4:training -2.9e+02 6.1e+02 3.8e+03 -0.48 0.63

in intercept:training 33 66 42 0.49 0.63

ns(Time, 4)1:in intercept:training -1.1e+02 26 3.8e+03 -4.4 1.3e-05

ns(Time, 4)2:in intercept:training -86 26 3.8e+03 -3.4 0.00074

ns(Time, 4)3:in intercept:training 37 51 3.8e+03 0.72 0.47

ns(Time, 4)4:in intercept:training -11 25 3.8e+03 -0.43 0.67
