# Supplementary material for: Augmenting complex and dynamic performance through mindfulness-based cognitive training: An evaluation of training adherence, trait mindfulness, personality and resting-state EEG
Source: PLoS One. 2024 May 20;19(5):e0292501. doi: 10.1371/journal.pone.0292501 (PMC11104625; doi:10.1371/journal.pone.0292501)
Supplement: Attachment — Submitted filename: Response_to_Reviewers.docx [file pone.0292501.s005.docx]

**PLOS ONE: PONE-D-23-29669**

**Response to Reviewers**

We thank the Reviewers for their helpful comments on our manuscript. Each comment has been carefully considered and significant changes have been made to the manuscript.

To summarise critical changes, we have:

1. Included additional details of the mindfulness intervention and EEG pre-processing procedures for transparency and replication

2 Justified sample choice (pertaining to size, age range and exclusion criteria)

3. Addressed concerns related to copyright and file naming conventions

Please find enclosed the revised manuscript, which includes changes intended to fully address the Reviewers’ comments. We have found the review process very valuable, and believe the manuscript is substantially improved.

All comments are shown in grey, while our responses are coded in black. All text snippets from the manuscript are *italicized,* with new content depicted in **bold**.

**Response to Editor**

1) Please ensure that your manuscript meets PLOS ONE's style requirements, including those for file naming. The PLOS ONE style templates can be found at [links provided].

We appreciate the editor’s provision of sample papers to amend the current manuscript’s formatting and file naming. To this end, we have reconfigured the title page, headings, and figure placements to align with PLOS ONE style requirements. Supplementary material titles and captions have also been added to the end of the manuscript file, with complementary – and separate - files attached.

2) Please provide an amended statement that declares *all* the funding or sources of support (whether external or internal to your organization) received during this study, as detailed online in our guide for authors at http://journals.plos.org/plosone/s/submit-now. Please also include the statement “There was no additional external funding received for this study.” in your updated Funding Statement. Please include your amended Funding Statement within your cover letter. We will change the online submission form on your behalf.

The funding statement within the paper has been revised to: *Preparation of this work was supported by a grant from the Defence Science and Technology Group [Research Agreement 9208] under the Research Network for Undersea Decision Superiority (RN-UDS). IB-S was supported by an Australian Research Council (ARC) Future Fellowship (FT160100437);* ***though the ARC did not provide any funding for this research. There was no additional external funding received for this study.***

3) In the online submission form, you indicated that your data will be submitted to a repository upon acceptance. We strongly recommend all authors deposit their data before acceptance, as the process can be lengthy and hold up publication timelines. Please note that, though access restrictions are acceptable now, your entire minimal dataset will need to be made freely accessible if your manuscript is accepted for publication. This policy applies to all data except where public deposition would breach compliance with the protocol approved by your research ethics board. If you are unable to adhere to our open data policy, please kindly revise your statement to explain your reasoning and we will seek the editor's input on an exemption.

We thank the editor for recognising our oversight in uploading data to the repository. In the endeavour to not hold up publication time, we have now uploaded all necessary data to the following repository: https://osf.io/y9hvt/?view_only=8adad97cc51841efb7e59fb2c92de970.

4) We note that Figure 1 in your submission contain copyrighted images. All PLOS content is published under the Creative Commons Attribution License (CC BY 4.0), which means that the manuscript, images, and Supporting Information files will be freely available online, and any third party is permitted to access, download, copy, distribute, and use these materials in any way, even commercially, with proper attribution. For more information, see our copyright guidelines: http://journals.plos.org/plosone/s/licenses-and-copyright.

We require you to either (1) present written permission from the copyright holder to publish these figures specifically under the CC BY 4.0 license, or (2) remove the figures from your submission:

a. You may seek permission from the original copyright holder of Figure 1 to publish the content specifically under the CC BY 4.0 license.
b. If you are unable to obtain permission from the original copyright holder to publish these figures under the CC BY 4.0 license or if the copyright holder’s requirements are incompatible with the CC BY 4.0 license, please either i) remove the figure or ii) supply a replacement figure that complies with the CC BY 4.0 license. Please check copyright information on all replacement figures and update the figure caption with source information. If applicable, please specify in the figure caption text when a figure is similar but not identical to the original image and is therefore for illustrative purposes only.

As suggested, we reached out to Neuropsychologia’s copyright team to ensure appropriate permissions for re-using Figure 1 within this manuscript’s publication. Their team has responded and stated that Elsevier does sign any 3^rd^ party forms, and its content cannot be published/considered as open access material. In this case, we have entirely re-created this figure to avoid the necessity of these copyright permissions (see below). Here, we refer to reader to the Neuropsychologia’s paper for a more detailed schematic.

**Fig 1.** **Schematic of the TMA simulation. This figure depicts a participant** engaging in the dual-screen TMA task within CRUSE. On the left monitor, participants are presented with a geoplot of their submarine, their trial tote (for adjusting contact parameters and setting new solutions) and current plotted solutions. On the right monitor, participants can view the dot stacks and compare their parameters with information received from SONAR, to assess the accuracy of their current solutions. For a more detailed diagram of the TMA task, please see (Dziego et al., 2023).

5) However, I have several concerns regarding the EEG:
1. Since the EEG is being explored in a resting condition, I consider it advisable to compare the EEG of each subject with the electroencephalographic norms to determine if they are within normal limits. Inclusion of participants with abnormal EEG features, often associated with neurological or psychiatric disorders, could severely bias the results.
2. In the Introduction it should be very clear, on the one hand, that the different recording conditions (rest, meditation, different types and difficulties of cognitive tasks) have characteristic EEG patterns that are different from each other. On the other hand, since from the resting EEG several study variables are derived, the reader should be informed what an increase (or decrease) in theta or alpha activity would represent in terms of a healthier state of intrinsic brain functioning. brain (e.g. For example, in the resting EEG is it desirable that theta power increases with the intervention?)

We agree that comparing the current study’s sample to norms would be beneficial to understanding the baseline expected levels of these resting-state metrics, and consequently, the magnitude of change parallel to mindfulness adherence. However, currently, we do not understand enough about these metrics to have explicitly defined norms. As the literature currently stands, there is no consensus on the exact individual alpha frequency or 1/f pattern that should be expected, say, at certain age ranges. While research is demonstrating some associations with various demographics or disorders (i.e., age, schizophrenia or sleep disorders; Cross, Kang, et al., 2022; Kurthen et al., 2020; Voytek et al., 2015), these relationships are only just surfacing. At this point in time, there is not enough information to adequately define concrete norms.

With this in mind - as is often recommended within EEG research to mitigate the variance associated with different conditions - we screened participants for neurological, cognitive, psychiatric and language disorders, as well as visual impairments, or medication-use that may impact EEG. Further, if we compare the current sample’s distribution of neural metrics to our other samples (i.e., Cross, Chatburn, et al., 2022; Cross, Kang, et al., 2022; Dziego et al., 2023) and more general literature in the area (Klimesch, 1999), they are very similar. Here, IAF ranges between 8Hz-11Hz and 1/*f* slopes between 0 and -2 are comparable to healthy participants in these alternative samples. Similarly, we are unable to comment on how increases in alpha/theta activity at resting-state would relate to healthier brain functioning; we can only summarise the observed relationships with performance and mindfulness training. Nevertheless, we have briefly noted the comparison of our neural metrics of interest to other samples within the manuscript (page 20): “**We note that these distributions are similar to comparative samples observed within the literature** [61, 69, 129, 130].”

We also thank the editor for their suggestion of more nuance in the explanation of previous EEG results, and agree that this will help readers to orient themselves within the current study’s resting-state focus. As suggested, we have briefly elaborated on some of the research included within the introduction to explicitly state where/when EEG has been examined (i.e., at rest, during meditation, during task engagement). For example:

(Page 7): “*While* ***oscillatory analyses of EEG during meditation*** *have commonly demonstrated increases in alpha power overall [50, 75], studies of IAF in particular have shown that* ***experienced meditators exhibit lower IAF during meditation*** *[76, 77*].”

(Page 6): “… *with previous literature demonstrating that* ***practicing*** *mindfulness meditation is most consistently associated with increases in power across both alpha and theta frequency bands (see [50, 51] for review)*.”

(Page 7): “*Previous work has also demonstrated that higher IAF* ***at rest*** *is associated with faster adaptation to complex and dynamic tasks [61].”*

(Page 7): “*Steeper slopes in this line* ***during rest*** *have been associated with increased performance on object recognition tasks [64], working memory tasks [60] and visuomotor tasks [63] as well as faster adaptation to novel language input [81].”*

We note that we have not aimed to investigate alpha/theta within the resting state, and thus have omitted elaboration of this within the paper.

6) If it had not been previously demonstrated that this task has no learning effect, this question should be addressed within the limitations of the study, i.e., to what extent certain tasks have a learning effect and, therefore, performance will be better in the 2nd. application. In this case, it is desirable that the authors make some recommendations for future studies.

As this is one of the first studies comparing TMA task performance across two sessions, we are unable to directly assess how these changes compare to changes across time without mindfulness practice. However, as noted within the paper, on average, participants are improving performance on the task in the second session. Nevertheless, there is large variability in participant’s change in performance from session 1 to session 2 (ranging from - 5272 to + 2145 TPE). Furthermore, with our within-groups design and dose-dependent adherence variable, we can somewhat capture what could be ‘practice effects’, where participants did not engage in the mindfulness sessions at all (i.e., adherence = 0). For example, extracting the model’s estimates when adherence = 0, gives a predicted TPE value of 5541 (on average across time). This suggests that the general practice effect would lead a participant to have a score of 5541, compared to engaging in ten mindfulness training sessions (resulting in predicted values of 4496 TPE) and sixteen mindfulness sessions (resulting prediction: 3914 TPE). As adherence was a significant predictor in the model, there is evidence to suggest that mindfulness predicts performance above practice effects. We have included a note of this proxy for practice effects in the results section (page 22, figure 6 caption): “**For comparative purposes, we note that as a general ‘practice effect’ (i.e., model predicted values when adherence = 0), participants demonstrate an average TPE of 5541 across experimental time**.”

7) HYPOTHESIS: According to what was mentioned in the Introduction regarding [60, 64], it seems contradictory to expect performance to improve on more complex and dynamic tasks and, simultaneously, to expect the negative slope of 1/f to increase.

We agree with the editor in that these particular resources support a hypothesis where a flatter 1/*f* slope is more likely to be associated with improved performance. Nevertheless, extant literature (i.e., Bornkessel-Schlesewsky et al., 2022; Donoghue et al., 2020; Immink et al., 2021; Ouyang et al., 2020) demonstrates more evidence towards steeper slopes relating to improvements in cognitive performance, and research within the neuroscience of mindfulness/meditation more generally suggest increased neural inhibition as a result of meditation (which is associated with steeper 1/*f* slopes; Gao et al., 2017). While we cannot be entirely clear on the expected change, our hypothesis aimed to be grounded in the predominant consensus within the literature. We have aimed to better capture this within the ‘current study’ section of the introduction, as follows: (Page 10) *“Thus,* ***in line with the majority of findings in the performance literature – alongside in meditation-related studies*** *- we expect to observe steeper 1/f slopes in individuals, in comparison to pre-intervention measures, following the mindfulness intervention. “*

**Response to Reviewer 1**

8) Clarification on Mindfulness Training Regime: The manuscript could benefit from more detailed information on the specific mindfulness techniques used, considering the variety of approaches within mindfulness practice.

We agree that the mindfulness intervention and protocol descriptions could be more clearly outlined and thus, we have added additional details within the manuscript. Please see comment 32 (below) for all additional information included within the methods and protocol.

9) Expansion on EEG Metric Analysis: While the manuscript addresses resting-state EEG metrics, further exploration into task-related EEG changes due to mindfulness training could provide additional insights.

We agree with the reviewer that additional analyses into task-related EEG changes could help to better elucidate the cognitive benefits gained from mindfulness training. Within the context of the current study, however, there are vast complexities to investigating the related on-task EEG data. For example, the unstructured nature of the task makes it difficult to time-lock to crucial elements. The submarine simulation also involves working across multiple screens, which would introduce large movement artefacts into the EEG signal (when compared to a more traditional, single-screen, static laboratory task). Moreover, our focus to date has been on investigating resting-state metrics in the context of cognitive performance (e.g. Dziego et al., 2023) and thus we aimed to extend on these findings in particular.

We note that we have captured that this is an excellent route for future research within the paper: *These findings highlight the need for future research to explore other avenues of neurophysiological change that may be underlying benefits from this style of cognitive training. For example, individuals may exhibit variations in oscillatory activity in-task (relating to attention or inhibition [148].*

10) Consideration of Long-Term Effects: The study focuses on short-term interventions. Including a follow-up assessment could provide valuable information on the longevity of the cognitive benefits observed.

We are encouraged by the interest in further elucidation of the long-term benefits of mindfulness training. While we have no doubt that the study would be enriched by the addition of a longitudinal follow up assessment, this was beyond the scope of the current protocol. We however want to reiterate the already novel nature of the study: this was one of the first experiments exploring more dynamic contexts and implementing only very brief interventions. We agree that a valuable next step within this area would be to assess longitudinal benefits of mindfulness-based cognitive training interventions, which we are beginning to see in pre-registrations online (e.g., Wong et al., 2016).

We have made a note of this in the discussion as follows: (Page 36) “*We look to future research to elucidate in more detail the ways in which distinct mindfulness practices work and* ***to better understand their benefits to cognition more longitudinally****.”*

11) Inclusion of a Control Group: The study could be strengthened by including a control group that does not receive mindfulness training to better attribute observed changes specifically to the mindfulness intervention.

As between-groups analyses of mindfulness interventions have been shown multiple times to improve performance across multiple studies/contexts, we opted to aim for more nuance in our study by investigating mindfulness practices as a within-subjects continuous factor. This analysis approach allows us to increase statistical power and assess dose-dependent effects of the intervention. In particular, dichotomisation of a variable can reduce power to the same extent as removing approximately a third of the data (Altman & Royston, 2006; Cohen, 1983). Additionally, we can better understand the underlying relationship between volume of mindfulness adherence and cognitive benefits while mitigating the potential issues evident with group assignment (and subsequent compliance/attrition confounds; as explored by Haynes & Dantes, 1987; Nam & Toneatto, 2016; Robiner, 2005).

12) Broader Participant Demographics: The participant age range is relatively narrow (18 - 38 years). Expanding this range could enhance the generalizability of the findings.

We agree with the reviewer that broader demographics would increase the generalisability of the findings. In our case, our demographic was specifically chosen for two reasons. As this project was funded by the Australian Defence Science and Technology (DSTG) group, our sample was inspired by real-world military applications, and aimed to be contextually relevant and reflective of individuals training within Defence. Furthermore, this age range helps to increase homogeneity of the sample, and mitigate variance within EEG data, as older adults exhibit age-related changes within their signals (e.g., Kurthen et al., 2020; Voytek et al., 2015). Again, we highlight the novel nature of the study on its own, representing a first step within this literature, but would encourage additional replication with other perspectives in mind (i.e., investigating lifespan changes) to solidify the findings. We have included this within our limitation and future direction section (page 36): “*We look to future research to elucidate in more detail the ways in which distinct mindfulness practices work* **and to better understand their benefits to cognition more longitudinally.”**

13) Addressing Potential Bias in Self-Reported Data: The study relies on self-reported adherence and enjoyment of the mindfulness practice. Consideration of potential biases in these reports should be discussed.

We thank the reviewer for bringing up this crucial consideration within mindfulness intervention studies. We agree that subjective reporting is a critical issue that can greatly impact findings from such research. However, this was recognised and considered during our experimentation, and so, while adherence was tracked subjectively (through the post-training surveys), we completed the final analyses using data gathered from objective tracking metrics (*Google Tag Manager*, and *Google Analytics*). To ensure there is no confusion, we have added an additional line to the mindfulness training methods (page 12): “***These objective metrics were used to identify individual participant adherence****.”*

With respect to mindfulness practice enjoyment, we think that this variable is best captured through subjective self-report of participants but do acknowledge the potential social desirability bias in this method. However, we hope to mitigate these effects by using online survey tools, in which participants do not directly report to the researcher. We have noted this within the manuscript as follows (page 31): “***We do acknowledge, however, that individual enjoyment reports may be influenced by social desirability bias. Nevertheless, it is hoped that these effects are mitigated by the use of online reporting tools, in which participants are not required to interact with the researcher.”***

**Response to Reviewer 2**

14) There are some major questions for this study, as well as the interpretation of the findings. Specifically, adherence, or compliance, to the mindfulness intervention was the primary variable in this study. Therefore, the main independent variable was operationally different than a week-long mindfulness intervention, rather it was a dose-dependent variable. Importantly, there was no post-analysis to determine if mindfulness actually increased from this intervention, so no validity/manipulation check was performed to measure the outcome itself of the mindfulness intervention (increased mindfulness). Therefore, the main predictor of outcomes may not be specific to mindfulness, per se, but rather the individual basis of whether or not a participant is compliant. This issue is further nuanced by the differential finding in that those who adhered to the training on the first day performed better in the TMA task. Overall, is the cognitive performance enhancement more related to compliance, or mindfulness? Can this be parsed out? Is this a spurious relationship? This needs to be further considered and discussed. Other comments are below.

We thank the reviewer for their careful consideration of internal validity of the study and the implementation of the intervention. We agree that it is not necessarily the mindfulness intervention per se, but the level of adherence to the regime that we are intending to measure here. With this in mind, we aimed to take a novel approach to traditional intervention studies and assess the dose-dependent effect of mindfulness techniques (as group-based effects have been demonstrated across multiple studies, see comment #11). Nevertheless, we wish to point out that the intention of this intervention was not to necessarily increase mindfulness. While mindfulness techniques are typically used with this goal, we wanted to instead investigate how a mindfulness intervention increased cognitive performance. Irrespective of whether ‘mindfulness’ truly increased or not (which as noted, we did not measure), the techniques that participants engaged in improved some aspect of cognition. As discussed by Malinowski et al. (2013), engaging in mindfulness practices may improve attentional mechanisms through repeated effortful attention regulation (monitoring distractions, re-orienting, sustaining), resulting in improvements to the brain networks of attention. While other studies may intend to measure how ‘mindfulness’ changed across the intervention, this was not the focus of the current study. Instead, we aim to see how these techniques can improve cognition, irrespective of their relationship to or effect on dispositional mindfulness. Here, the next steps would be to further understand the mechanisms of this – whether it is increasing ‘mindfulness’ or simply developing attentional capacity as a result of these practices.

For the issue of compliance, we interpret this comment as inquiring about fundamental differences between our lower and higher adherers. Firstly, our mixed models controlled for baseline performance, in the endeavour to account for fundamental differences in our participants at session 1. Even while controlling for baseline performance, those who adhered to training earlier on, showed greater performance in the second testing session. We feel that this timing analysis helps to elucidate how *timing of adherence*, and not just overall adherence (or compliance) particularly highlights the salutary effects of mindfulness training. Here, irrespective of compliance in later sections of the intervention (i.e., in the second half of the week), all earlier engagement in mindfulness training was associated with greater performance, suggesting that the engagement in the mindfulness - not necessarily compliance to overall instructions - is driving the effect. Additionally, as a proxy for compliance, we believe that something like conscientiousness could be representative, however, there were no relationship between this and adherence (R^2^ = -0.0096, *p* = 0.95).

15) The authors do a nice job of pointing out that mindfulness has not consistently been found to increase cognitive performance, and suggest this is due to individual differences. I suggest the authors also include a discussion of how mindfulness itself as a variable may contribute to these differences, and discuss in which context mindfulness may be more likely to influence cognitive outcomes. For example, did these studies look at mindfulness as a trait, a brief mindfulness session, long term mindfulness training, etc? This is important as the current study implements a somewhat ‘in-the-middle’ approach between an acute session and long-term training.

We agree that it is critical to delineate where and how mindfulness was used across the studies mentioned within the introduction. As suggested, we have attempted to more clearly highlight where mindfulness is defined as an intervention, versus a trait variable, when summarising previous findings. For examples, see as follows:

*(Page 5): “Even* ***engaging in*** *very brief interventions (20 minutes each day for four days) improve visuo-spatial processing, working memory and executive functioning [24] and a single session of focused attention monitoring* ***immediately*** ***prior to learning*** *can improve consolidation of motor patterns [18, 27].”*

*(Page 5): “Similar* ***brief pre-test engagement in mindfulness techniques*** *have reduced inattentional blindness [28], improved Stroop Test scores [29], increased flanker task performance [30] and working memory ability [31].”*

We note that we have previously discussed how mindfulness as a trait may influence intervention outcomes and cognitive performance within the introduction (page 6): *“In particular, levels of dispositional (or trait) mindfulness refer to intra-individual differences in the frequency with which individuals experience subjective awareness to the present moment (i.e., frequency of ‘mindful states’; [39], which has been shown to interact with outcomes of mindfulness-based interventions in depression [40] and within cognitive testing contexts [41]. Moreover, trait mindfulness has been shown to positively correlate with self-reported attentional capacity [42], suggesting that it is a necessary consideration within investigations of cognitive performance.”*

Regarding the large heterogeneity in contextual factors across mindfulness-cognition research, (e.g., in the amount of training, consideration of trait mindfulness and populations examined), we note that there is prevailing homogeneity in the findings and outcomes of mindfulness interventions. In this case, we have added the following sentence to summarise this explicitly to our readers (page 4): “***Overall, even with the large heterogeneity in interventions, engaging in some type of mindfulness practice (whether short-term, longer term or single session modalities) appears to be beneficial to cognitive functioning****.”*

Furthermore, we appreciate the reviewers’ acknowledgement of our novel approach to the intervention study with an ‘in-the-middle’ and more time practical approach. To also make this clearer to readers, we have aimed to better describe our approach within the current study section, noting that (Page 9): *“****Here, we used adherence to the mindfulness intervention as a continuous dependent variable. In this case, the study hopes to better capture how true engagement in the mindfulness practices (not just group assignment; and often, varied compliance), relates to performance enhancements****.”*

16) The understanding of direction of alpha and theta activity are fundamentally different, so please explain in the broader sense what this statement implies/means: …”increases in alpha and theta oscillatory activity (see [50] for review).” – line 2, pg 6

Here, we are summarising findings from analyses of oscillatory activity in mindfulness research. In particular, this sentence aims to re-state the conclusions from the Lotas et al. (2015) and Cahn & Polich (2006) review, in which power within the alpha and theta frequency ranges commonly appears to increase *during* meditation across a range of studies. We do agree that this is not clearly specified within this statement, and have thus amended as follows:

*“The neuroscientific exploration of mindfulness has primarily focused on the investigation of on-task (or evoked) neural dynamics, with previous literature demonstrating that mindfulness meditation is most consistently associated* ***with increases in power across both alpha and theta frequency bands*** *(see [50, 51] for review).”*

However, as this is not the focus of the study and is included to simply showcase the focus of previous research, we have opted to avoid elaborating on the distinction and the functional relevance of neural activity within the alpha and theta frequency bands within the manuscript.

17) This sentence is incomplete: “from mindfulness practice, as well as distinguishing “ – line 7 page 6

We have amended this section which now currently reads as (page 6): “*While resting-state activity is often explored in the context of neurological disorder or dysfunction (e.g., [54–58]), it has also been investigated in relation to peak cognitive performance [59–63] and thus may be able to provide insights into salutary neuroplastic changes from mindfulness practice.”*

18) Page 6, please explain alpha in more detail (it’s stated generically as “alpha band” – explain what alpha band is and what this means). Similarly, alpha power and alpha frequency are not the same but reads as if they are in this paragraph (2 on page 6) of the paper. This suggestion follows true for other frequency bands mentioned in the intro (theta, gamma, and beta).

We appreciate that the distinction between alpha band and frequency was not clear in our initial explanation of IAF and thank the reviewer for encouraging clearer communication within our introduction. In this case, we have clarified the distinction by better describing IAF as follows:

*Two resting-state characteristics of electroencephalography (EEG) recordings that have been associated with cognitive functioning and performance include aperiodic 1/f activity [60, 63-66] and the individual alpha frequency (IAF) [62, 67, 68].* ***IAF refers to the frequency (Hz) of maximal neural power (uV) within the alpha band range (7.5-12.5Hz; (Klimesch, 1999)****. IAF is typically estimated during an eyes-closed resting state and has been found to positively correlate with IQ [62], visuo-perceptual ability [68, 70, 71] and memory performance [67, 72]. More recent findings are demonstrating that IAF might not simply represent enhanced global cognition, but may indicate individual differences in information processing [73, 74]. Previous work has also demonstrated that higher IAF is associated with faster adaptation to complex and dynamic tasks [61].* ***While oscillatory analysis of EEG during meditation have commonly demonstrated increases in alpha power overall*** *[50, 75], studies of IAF* ***in particular*** *have shown that experienced meditators exhibit lower IAF during meditation [76, 77]. However, whether these changes persist long-term (altering the resting-state) and how they relate to enduring cognitive benefits is unknown.*

19) Line 28 on page 6 needs revision: “…various experimentation…”

The sentence has been amended to improve clarity and now reads as follows (page 6)*:* “*More generally, aperiodic 1/f activity is largely thought to reflect the excitation/inhibition balance within neural networks [82, 83]. This notion has been* ***supported by studies exploring the influence of*** *alcohol [84], propofol [85], sleep [78, 86] and psychiatric disorders [57, 58, 87]* ***on the 1/f slope.”***

20) Page 7: I’m not sure if this is accurately described as a theory? “..possible that steeper 1/f slopes may be observed long-term following brief/sustained mindfulness practice. Consistent with this theory, …”

As suggested, we are not intending to discuss an over-arching cognitive theory or framework here. In this case, we have amended the sentence as follows (page 8): “*Consistent with this* ***notion****, meditation interventions have been successfully employed in the prevention of age-related declines in cognition [89, 90], suggesting that meditation may impede age-related flattening of the 1/f slope [91].”*

21) Line 13 on page 7 says “practitioners”, but I think you don’t mean this word (as in a clinician?)?

While we understand that the word ‘practitioner’ is commonly linked to doctors or clinicians, this word is also used within the meditation and mindfulness literature to refer to individuals who are experienced meditators and actively engaged in their practice. For examples within meditation and cognitive neuroscience literature, see Brefczynski-Lewis et al. (2007), Fox et al. (2014) and Luders et al.(2011). In this case, we have chosen to keep this phrasing within the current manuscript.

22) Another possible incorrect word choice used for “equivocal” in line 23 of page 7

To ensure better clarity within the writing, this sentence has been revised to (page 7): *However, findings* ***have not been entirely straight-forward****, suggesting that unaccounted factors (e.g., levels of adherence and individual factors) might moderate these effects.*

23) Please explain what you mean here; “…in the effort to provide more nuanced findings.” Is there a benefit to providing more nuanced findings? This would suggest you are trying to find more variability, more complexities, as opposed to getting more directly at the relationship you are investigating.

We agree with the reviewer that while our study attempts to account for more nuance in participants, contexts and techniques, the underlying aim is to provide more clarity and less complexity to the final findings/results. In this case, we have altered this statement to read (page 8): *The current study therefore aims to investigate adherence to mindfulness-based cognitive training and individual differences, alongside performance-based enhancements in cognition, in the effort to provide* ***more clarity*** *to the findings.*

24) You bring up low dimensional cognitive tasks, what does this mean exactly? The examples provided are mostly examples of executive functioning tasks, which would assume to translate to real-world applications. Please clarify.

When referring to cognitive tasks of low dimension, we aim to describe the relatively poor ecological validity of computerised cognitive tests. There is still a large gap between typical in-lab cognitive task versus the real-world complexity of human-environment interaction, whether this is because of their predictive nature or controlled environment. However, we understand that the term ‘low-dimensional tasks’ may not directly capture our intended meaning and have thus aimed to clarify this phrase by, instead, using (page 3): ‘*narrow nature of cognitive tasks employed’*.

.

25) What are ‘mutable’ tasks? Line 2 page 8.

‘Mutable’ tasks refer to tasks that are more unpredictable, fluctuating and variable, which better epitomise the complexities of human-environment interaction in the real world.

26) In page 8, findings by Brown et al are the rational for an exploratory analysis, but this study is not mentioned prior to this statement. In general, the summary/concluding paragraph(s) of the introduction provides new content that was not previously supported or provided as rational for the purpose or hypotheses of the current study.

We agree with the reviewer that it is important to contextualise these findings prior to the final paragraph of the introduction. We note that we have included a mention of this study within the earlier paragraphs on the introduction, but failed to make the link clear by directly referring to the Brown et al. study. In this case, we have amended this initial mention to include this (page 5): “*Here, such investigation could help to differentiate whether mindfulness-based training might be more beneficial as a ‘consolidation’ practice (e.g., helps to improve memory of the task instructions from the first session; theorised by* ***Brown et al.*** *[35]) versus an attentional/state enhancing tool (e.g., helps to create an optimal attention state for task performance in the second session; as demonstrated by single session, brief training enhancements [18, 28, 31, 36].”*

Furthermore, we have opted to make the link clearer within the final paragraph of the introduction by rephrasing the connecting sentence. This is as follows (page 10): *As an additional exploratory analysis, motivated by findings in Brown et al. [35] - where post-training mindfulness expedited wakeful* ***consolidation*** *of motor sequences - we examined how adherence to the training protocol prior to sleep might influence its direct effects on performance, irrespective of total overall volume.*

27) Please revise sentences throughout for word choice/meaning/clarity. For example, a hypothesis can’t be “determined” (a hypothesis is stated, not determined) that variables “may” (suggestive wording is not correct in a hypothesis) interact.

As suggested by the reviewer, we have amended particular word choices for clarity. Here, the sentence has been changed as follows (page 10): “*In particular, we* ***predicted*** *that initial levels of dispositional mindfulness* ***would*** *interact with benefits gained from the mindfulness-based cognitive training regime.* *Higher levels of neuroticism, and lower levels of conscientiousness,* ***should*** *be negatively related to adherence. Further, resting-state metrics may provide insight into individual capacity to optimally engage in mindfulness practices (and thus derive benefits), or could help to track neural changes (associated with improved performance) following mindfulness interventions. In this instance,* ***we expect to observe*** *steeper 1/f slopes in individuals, in comparison to pre-intervention measures, following the mindfulness intervention.”*

28) How many participants did you have to exclude if they reported history of neurological, cognitive, psychiatric or language disorders? Also, in the discussion or limitations section it would help to speak to the generalizability of this sample. It would possibly help also to be specific on what the inclusionary criteria were, and specifically the exclusionary criteria. I’m assuming some of this was exclusionary, as the recruitment aspect particularly did not bias participant recruitment by including reference to meditation or mindfulness.

Prior to any involvement in the study, all participants were screened for histories of neurological, cognitive, psychiatric or language disorders. While a small number of individuals did not meet these criteria upon their screening, they did not participate in any part of the study prior to exclusion and we consequently have no record of them. To make this clear, the method section has been amended as follows (page 11): “*To participate,* ***individuals were screened*** *and must have reported no history of neurological, cognitive, psychiatric or language disorders.”* See comment #12 by Reviewer 1 for further information on the generalisability of the sample.

29) For the sample size, please justify the power of this sample for statistical analyses. It appears to be underpowered.

For responses to this comment and similar, see comment #39.

30) What exactly was the personality questionnaire? Was this a validated measure of the personality traits you measured? If not the issue of construct validity needs to be addressed.

We thank the reviewer for bringing this absence of information to our attention. We agree that additional descriptions of the personality questionnaires would be valuable to demonstrate validity and aid in replicability of the study. In this case, we have expanded on the personality questionnaire section, as shown below (page 12):

*Questions* ***originated from the short-form International Personality Item Pool (IPIP [102-104]) and*** *aimed to target the two Big 5 [43] traits of interest (neuroticism and conscientiousness).*

31) For measures, were there any missing data for any of the items in each of the surveys/questionnaires and if so how was this missing data addressed since the questionnaires were summed for a total score? Following this same notion, were there any participants that did not complete a specific portion of the study?

We appreciate the reviewers’ caution around missing data and consistency in scoring the surveys and questionnaires. As we presented our surveys through *Qualtrics*, we were able to enforce a forced-choice paradigm, in which participants could not move through to subsequent questions if prior questions were not answered. This ensured no missing data for personality and demographic questionnaires. We have added an additional comment in the personality questionnaire section (page 11) to address this: “***To ensure that participants responded to all items within the questionnaire, we enforced a force-choice paradigm, in which the next question was only presented once the prior section was answered.”***

Nevertheless, we did encounter variation in the quantity of responses for self-reported ‘enjoyment’ of the exercises. This survey was completed independently by the participant after their final session each day and therefore the number of responses greatly varied in relation to their level of adherence. We have however, described how this was addressed within the paper (page 13): “*We note that some participants did not always engage in their prescribed mindfulness session (or complete their surveys) and thus scores were created from any potential responses provided (range 1 – 9 responses)”.* See comment #32 for discussion on the range.

32) During the mindfulness intervention and protocol descriptions, please provide a little more detail. For example, could participants do all three 5-minute mindfulness sessions in a row, or did they have to be separated by a certain amount of time? Also, it is stated there were seven distinct recordings, what determined the recordings that were chosen? Did it matter if they listened to the same one each time? Additionally, was the same type of mindfulness implemented in each recording, or was this variable? How exactly were participants encouraged to complete the post-training questionnaire? Did this occur each day, only at the beginning, etc?

We thank the reviewer for their suggestion to clarify the mindfulness intervention and protocol descriptions, to better provide other researchers opportunities to replicate the study’s design. Alongside with recommendations from Reviewer 1 (see comment #8), we want to make the following clarifications and present the changes we have made to the methods section below:

- Included information about mindfulness sessions being completed whenever best fits the participant’s schedule. While participants were encouraged to not do the exercises in a row (and space them out throughout the day), they could complete the exercises whenever best suited them (either in a row, or up to three separate times throughout the day).
- Audio recordings were presented to participants through separate web page tabs per day (i.e., day 1, day 2, etc.), with each day including one recording. This means that when participants did three sessions per day, they listened to three of the same recording.
- The types of mindfulness presented to participants varied across the seven days. Specifically, day 1 involved simply observing and focusing on breathing, and then on day 2, the whole body (in the style of focused attention meditation). Day 3 involved a body scan, again, in the style of focused attention meditation, whereby attention was directed sequentially across different areas of the body. The day 4, day 5 and day 6 audio recordings comprised of focusing attention on observing and counting the breath, but began to introduce concepts within open monitoring meditation: observing when distractions arise. In day 6, the guided mindfulness session involved expanding attention to monitoring the entire body through breath, and finally, day 7 focused on open monitoring techniques, where attention is expanded to experiencing external surroundings.
- A link to the post-training survey was presented on each daily page, alongside the following information: “we ask that at the end of your last exercise each day, you fill out a short questionnaire of your experience. You can find these on each daily page”. We note that this is already specific within the manuscript: *Following their last session each day, participants were encouraged to complete a ‘post-training’ questionnaire about their experience and how many times they were able to partake in the sessions (see post-training survey below).*
- To assist with ensuring participants felt comfortable completing the exercises, there was a frequently asked question page (clarifying ‘am I doing this right?’ and ‘how am I supposed to feel?’).
- As stated in the paper, we also reiterate that all webpages and audio tracks can be viewed on the github repository: <https://github.com/chloeadee/cognitivetraining>

With these in mind, the following has been added to the mindfulness-based cognitive training intervention section (page 11):

***A web-browser based online*** *mindfulness-training program was implemented through short audio recordings voiced by an experienced mindfulness instructor (****based on mindfulness techniques previously used in research*** *[35, 105, 106]). Seven distinct audio tracks (approximately 5 minutes in length) were recorded for the current study****. All transcripts and audio recordings can be accessed by the Open Science Framework (OSF) repository here: https://osf.io/y9hvt/.*** *These recordings guided participants through a variety of focused attention and open monitoring mindfulness practices* ***(for full descriptions of these techniques, see [2, 107]),*** *using cues such as “direct your attention to your breath”.* ***Audio recordings on day 1 and day 2 involved observing and focusing on breathing or the body, while day 3 expanded to sequentially moving attention across the entire body (in the style of focused attention meditations). Day 4, 5 and 6 comprised of focusing attention on observing and counting the breath but began to introduce concepts of open monitoring meditations (i.e., observing when distractions arise). Audio recordings for days 6 and 7 gradually instructed more open monitoring techniques, where attention was expanded to noticing thoughts and non-judgmentally experiencing external surroundings.***

*Participants were able to access the audio recordings* ***through their web-browser directed*** *to an online repository hosted on GitHub Pages (GitHub Inc., 2021; accessible at https://github.com/chloeadee/cognitivetraining****). Each recording was accessed individually on a daily basis based on the layout of the web-page****. HTML code for the webpage was produced using RMarkdown in R [108]. Instructions on the webpage encouraged participants to engage in the brief mindfulness sessions three times per day; preferably in separate sessions (i.e., three exposures to the same 5 minute recording; for a total of 15 minutes each day). Following their last session each day,* ***the website prompted participants*** *to complete a ‘post-training’ questionnaire about their experience and how many times they were able to partake in the sessions (see post-training survey below). Unbeknownst to participants, training adherence was additionally tracked using Google Analytics and Google Tag Manager (Google Inc., 2021). Tracking metrics recorded when and how many times participants accessed the site’s various pages and completion of a mindfulness session was counted when audio tracks were played for > 90% of their duration.* ***These objective metrics were used to identify individual participant adherence.***

33) Also, please clarify what you mean by the last sentence in paragraph 1 on page 11. It sounds like there would have been a max total of 7 responses per participant, but the range is 1 – 9. I am not clear on what this range therefore pertains to.

We thank the reviewer for bringing this to our attention. It is true, that in fact, the maximum number of responses here should be 7 for seven days of cognitive training. However, three participants completed this questionnaire multiple times on one day, creating a maximum response of 9. To avoid confusion, this has now been explicitly stated within the study:

*We note that some participants did not always engage in their prescribed mindfulness session (or complete their surveys) and thus scores were created from any potential responses provided (range 1 – 9 responses;* ***three participants provided more than one response per day, resulting in a maximum of 9 responses****).*

34) In EEG data analysis, please provide more details. For example, there is no mention of artifact rejection, impedance levels, how were eye blinks handled, how were right/left eye movements measured and handled, etc? Also, please provide rationale for high pass filter of 1 Hz. It was recently suggested this filter setting not go above .9 Hz for most studies, as the noise ratio greatly increase with higher filtering setting (below), so please address this. (Zhang G, Garrett DR, Luck SJ. Optimal Filters for ERP Research II: Recommended Settings for Seven Common ERP Components. bioRxiv [Preprint]. 2023 Jun 14:2023.06.13.544794. doi: 10.1101/2023.06.13.544794. PMID: 37397984; PMCID: PMC10312706.)

We acknowledge that we do not mention many of the pre-processing steps found in conventional on-task EEG analyses. We agree that mention of impedance levels is critical, and we have thus added this into the EEG recording section (page 15): *“****Impedances were kept below 10kΩ****.”* Otherwise, information about artifact rejection, blinks and movements is absent as these pre-processing steps were not completed in the current study. As we use two minutes of EEG recordings - where the participants sit completely still, with eyes closed - many of the conventional steps (ICAs, etc.) are superfluous. For example, there are no blinks to be identified. Regarding the 1Hz filter, we note that we are not interested in activity below this range (as is common in ERP research). In addition to this, these pre-processing steps are consistent with our previous work in this area (e.g., Dziego et al., 2023) to ensure that results are comparable.

35) How many people dropped out between time point one and two? It was stated 2 participants dropped out, so is this the attrition between time points?

This is correct. Within our sample, two participants completed only one session, and therefore, no outcome variables were recorded. Consequently, all corresponding data was removed from the dataset. The final forty participants completed all components of data collection. This has been noted within the participant section.

36) Figure 2 mentions data was log transformed, as typical in power analyses, but this is not mentioned in the methods.
37) Please expand on the analyses used from Corcoran et al (2018).

We appreciate the reviewer’s attention to detail and acknowledge the absence of finer details of the Corcoran et al. (2018) procedure. To ensure that readers who are unfamiliar with the technique can understand the full process, we have amended this section as follows (page 16):

*Individual alpha frequency (IAF) was calculated in line with procedures outlined in Corcoran et al., (2018). IAF was calculated from seven occipital-parietal electrodes (P3/P4/O1/Oz/O2/P7/P8) using philistine.mne.savgol_iaf (Alday, 2019) in MNE-Python [109].* ***These electrodes were chosen as the individual alpha peak is most predominantly discernible across these areas****. This method uses a Savitzky-Golay filter (frame length = 11 frequency bins, polynomial degree = 5 [112]) to smooth the power spectral density (PSD) before determining the peak of activity within the defined frequency range (in this case, 7-13 Hz).* ***Here, PSD estimates are gathered from fast Fourier-transformed EEG signals and represent the power of neural activity (uV^2^) within each frequency.*** *This method calculates IAF using both Peak Alpha (PAF) and Centre of Gravity (CoG) techniques.* ***For a full description of the technique, see [110].***

38) Why were occipital-posterior electrodes used for measure of interest? Typically, frontal electrodes are used when measuring cognitive-related activity.

While frontal electrodes are commonly used in the investigation of on-task oscillatory activity related to cognition, our resting-state metric approach represents a departure from traditional EEG power analyses. Resting-state metrics are more global and aim to capture largescale characteristics of the brain’s overall electrical activity. In this case, we have chosen to use occipital-posterior electrodes as the peak alpha frequency is most predominantly discernible over these areas while eyes are closed (Corcoran et al., 2018). While the individual alpha frequency peak can be seen across all areas of the scalp, the peak most clearly manifests across occipital areas, thus generating the cleanest data for individual alpha frequency estimation.

Moreover, for 1/*f* activity, parametric characterisation of this metric across the scalp shows little variation, and does not yet have particular topographical recommendations. As demonstrated in Figure 5, and analyses in previous literature (e.g., Ouyang et al., 2020; Voytek et al., 2015), the distribution of 1/f and cognitive outcomes is consistent irrespective of topography. With this in mind, we chose to use to occipital-posterior electrodes for consistency in our resting-state metric estimation approach. We hope the additional inclusion of more details of the Corcoran et al., 2018 analysis recommendations helps to also better highlight the differential nature of our resting-state approach.

39) This model is underpowered with only 40 participants: = 0 + 1 ∗ 2h ∗ 3 + 4 + 5 + 0 +

We acknowledge the reviewer’s concerns regarding the robustness of our model's power. However, we would like to highlight several points to strengthen the rationale behind our sample size and modelling choices. Firstly, it's important to note that the absence of standardized guidelines for conducting formal power analyses for mixed models makes it difficult to determine a study’s power in the same way that is typically done for more traditional modelling methods.

We ran a suggested method for power analysis in mixed models using the *simr* package (Green & MacLeod, 2016), using an effect size of 0.34. A recent meta-analysis by (Cásedas et al., 2020) reported that the effect of mindfulness-based cognitive training interventions on executive functioning scores typically yield a Hedge’s g value of 0.34, while a multi-study investigation of mindfulness training on working memory performance noted that “future studies are expected to have a ΔSMC ranging from 0.33 to 0.73” (Jha et al., 2022). For our simplest model, this simulation stated that there was 100% power for Time x Adherence interaction.

Previous work examining the impact of mindfulness on EEG parameters typically involve participant numbers ranging from 16 to 25 (Cahn et al., 2010; Lu & Rodriguez-Larios, 2022), with group-based analyses conducted with 29 and 22 participants per group (Rodriguez-Larios et al., 2021; Saggar et al., 2012). Our study's repeated measures design also captures performance metrics at 20-second intervals, yielding over 90 observations for each participant (totalling 3820 observations overall). This aligns with the ‘rule of thumb’ for mixed models, whereby each level of random effect should include a minimum of 20-30 observations. Moreover, our decision to treat adherence as a continuous variable rather than using a group-based analysis enhances the statistical power of our study, as supported by literature (Altman & Royston, 2006; Cohen, 1983). We also re-ran the simplest version of our models (i.e., TPE ~ time * adherence + (1 | participant), excluding the two covariates: decline and baseline) and found that all significant effects remained stable.

40) Some participants’ performance decreased in the TMA task at time point 2 – was this group analyzed separately and compared to those that increased their performance? Wondering if there is a fundamental difference between these participants. Also, did those that decrease their performance have a difference in their adherence?

To not decrease power within our analyses, this group was not separately analysed from those that increased performance. As discussed above (comment #14), we believe that these differences are best explained by adherence, and not fundamental differences in our participants. Like any cognitive performance study, there is large variability in how performance can change within an individual participant over time. For example, even with sleep deprivation, not all people will perform worse; some will perform better than their baseline (Belenky et al., 2003). Within linear mixed models, using random effects can help account for these individual differences (Bates et al., 2015). Our models also controlled for session 1 performance, which should have accounted for fundamental differences between participants, when predicting how adherence related to performance. We believe that this question taps into exactly what we are trying to capture within our study, in that decreases in performance are associated with less adherence.

41) Overall discussion section is well done. Conclusions would be addressed pertaining to the initial paragraph of this review.

We are pleased to hear this from the reviewer. In lieu of our discussions around improving mindfulness versus improving cognitive performance *through* mindfulness (see comment #14), we have chosen to just briefly touch on this within the discussion section. We have amended the following sections to better capture the focus of our study, and to align with our rebuttal, suggesting that mindfulness training, and not mindfulness trait increases, are associated with cognitive benefits in dynamic tasks:

(Page 35): “***Similarly, while the study highlights the intricate relationship between mindfulness interventions and cognitive enhancement, we did not directly examine whether participants showed higher levels of dispositional mindfulness following the intervention. Future approaches should aim to thoroughly examine mindfulness’ differential impacts on cognition, trait mindfulness, and subsequent inter-relationships.*** *We note that, overall, this study aimed to be more exploratory in nature, in that we investigated outcomes in more dynamic and complex settings. However, we look to future research to elucidate in more detail the ways in which distinct mindfulness practices work and* ***to better understand their benefits to cognition more longitudinally.”***

(Page 38): “***In summary****, the present findings* ***illustrate the propensity of mindfulness-based trainin****g to transfer cognitive* ***enhancements to performance on an unrelated complex and dynamic task****.* ***Practically, the present work has demonstrated an effective solution to implement an online mindfulness-based training program through a web browser approach, affording objective monitoring of individual training adherence.”***

**References**

Alday, P. M. (2019). *Philistine* [Computer software]. https://philistine.readthedocs.io/en/latest/api/philistine.mne.savgol_iaf.html

Altman, D. G., & Royston, P. (2006). The cost of dichotomising continuous variables. *BMJ : British Medical Journal*, *332*(7549), 1080.

Bates, D., Mächler, M., Bolker, B., & Walker, S. (2015). Fitting Linear Mixed-Effects Models Using lme4. *Journal of Statistical Software*, *67*(1), Article 1. https://doi.org/10.18637/jss.v067.i01

Belenky, G., Wesensten, N. J., Thorne, D. R., Thomas, M. L., Sing, H. C., Redmond, D. P., Russo, M. B., & Balkin, T. J. (2003). Patterns of performance degradation and restoration during sleep restriction and subsequent recovery: A sleep dose‐response study. *Journal of Sleep Research*, *12*(1), 1–12. https://doi.org/10.1046/j.1365-2869.2003.00337.x

Bornkessel-Schlesewsky, I., Sharrad, I., Howlett, C. A., Alday, P. M., Corcoran, A. W., Bellan, V., Wilkinson, E., Kliegl, R., Lewis, R. L., Small, S. L., & Schlesewsky, M. (2022). Rapid adaptation of predictive models during language comprehension: Aperiodic EEG slope, individual alpha frequency and idea density modulate individual differences in real-time model updating. *Frontiers in Psychology*, *13*, 817516. https://doi.org/10.3389/fpsyg.2022.817516

Brefczynski-Lewis, J. A., Lutz, A., Schaefer, H. S., Levinson, D. B., & Davidson, R. J. (2007). Neural correlates of attentional expertise in long-term meditation practitioners. *Proceedings of the National Academy of Sciences*, *104*(27), 11483–11488. https://doi.org/10.1073/pnas.0606552104

Cahn, B. R., Delorme, A., & Polich, J. (2010). Occipital gamma activation during Vipassana meditation. *Cognitive Processing*, *11*(1), 39–56. https://doi.org/10.1007/s10339-009-0352-1

Cásedas, L., Pirruccio, V., Vadillo, M. A., & Lupiáñez, J. (2020). Does Mindfulness Meditation Training Enhance Executive Control? A Systematic Review and Meta-Analysis of Randomized Controlled Trials in Adults. *Mindfulness*, *11*(2), 411–424. https://doi.org/10.1007/s12671-019-01279-4

Cohen, J. (1983). The Cost of Dichotomization. *Applied Psychological Measurement*, *7*(3), 249–253. https://doi.org/10.1177/014662168300700301

Corcoran, A. W., Alday, P. M., Schlesewsky, M., & Bornkessel‐Schlesewsky, I. (2018). Toward a reliable, automated method of individual alpha frequency (IAF) quantification. *Psychophysiology*, *55*(7), e13064. https://doi.org/10.1111/psyp.13064

Cross, Z. R., Chatburn, A., Melberzs, L., Temby, P., Pomeroy, D., Schlesewsky, M., & Bornkessel-Schlesewsky, I. (2022). Task-related, intrinsic oscillatory and aperiodic neural activity predict performance in naturalistic team-based training scenarios. *Scientific Reports*, *12*(1), Article 1. https://doi.org/10.1038/s41598-022-20704-8

Cross, Z. R., Kang, T. Y., Lushington, K., Sarkar, P., Singh, P., Johnston, S., Yeo, A., & Chatburn, A. (2022). *Resting-state aperiodic neural activity as a novel objective marker of daytime somnolence* [Preprint]. Neuroscience. https://doi.org/10.1101/2022.08.07.503118

Donoghue, T., Haller, M., Peterson, E. J., Varma, P., Sebastian, P., Gao, R., Noto, T., Lara, A. H., Wallis, J. D., Knight, R. T., Shestyuk, A., & Voytek, B. (2020). Parameterizing neural power spectra into periodic and aperiodic components. *Nature Neuroscience*, *23*(12), Article 12. https://doi.org/10.1038/s41593-020-00744-x

Dziego, C. A., Bornkessel-Schlesewsky, I., Jano, S., Chatburn, A., Schlesewsky, M., Immink, M. A., Sinha, R., Irons, J., Schmitt, M., Chen, S., & Cross, Z. R. (2023). Neural and cognitive correlates of performance in dynamic multi-modal settings. *Neuropsychologia*, *180*, 108483. https://doi.org/10.1016/j.neuropsychologia.2023.108483

Fox, K. C. R., Nijeboer, S., Dixon, M. L., Floman, J. L., Ellamil, M., Rumak, S. P., Sedlmeier, P., & Christoff, K. (2014). Is meditation associated with altered brain structure? A systematic review and meta-analysis of morphometric neuroimaging in meditation practitioners. *Neuroscience & Biobehavioral Reviews*, *43*, 48–73. https://doi.org/10.1016/j.neubiorev.2014.03.016

Gao, R., Peterson, E. J., & Voytek, B. (2017). Inferring synaptic excitation/inhibition balance from field potentials. *NeuroImage*, *158*, 70–78. https://doi.org/10.1016/j.neuroimage.2017.06.078

Green, P., & MacLeod, C. J. (2016). SIMR: An R package for power analysis of generalized linear mixed models by simulation. *Methods in Ecology and Evolution*, *7*(4), 493–498. https://doi.org/10.1111/2041-210X.12504

Haynes, R. B., & Dantes, R. (1987). Patient compliance and the conduct and interpretation of therapeutic trials. *Controlled Clinical Trials*, *8*(1), 12–19. https://doi.org/10.1016/0197-2456(87)90021-3

Immink, M. A., Cross, Z. R., Chatburn, A., Baumeister, J., Schlesewsky, M., & Bornkessel-Schlesewsky, I. (2021). Resting-state aperiodic neural dynamics predict individual differences in visuomotor performance and learning. *bioRxiv*, 2021.04.08.438941. https://doi.org/10.1101/2021.04.08.438941

Jha, A. P., Zanesco, A. P., Denkova, E., MacNulty, W. K., & Rogers, S. L. (2022). The Effects of Mindfulness Training on Working Memory Performance in High-Demand Cohorts: A Multi-study Investigation. *Journal of Cognitive Enhancement*, *6*(2), 192–204. https://doi.org/10.1007/s41465-021-00228-1

Klimesch, W. (1999). EEG alpha and theta oscillations reflect cognitive and memory performance: A review and analysis. *Brain Research Reviews*, *29*(2), 169–195. https://doi.org/10.1016/S0165-0173(98)00056-3

Kurthen, I., Meyer, M., Schlesewsky, M., & Bornkessel-Schlesewsky, I. (2020). Individual Differences in Peripheral Hearing and Cognition Reveal Sentence Processing Differences in Healthy Older Adults. *bioRxiv*, 2020.06.16.118943. https://doi.org/10.1101/2020.06.16.118943

Lu, Y., & Rodriguez-Larios, J. (2022). Nonlinear EEG signatures of mind wandering during breath focus meditation. *Current Research in Neurobiology*, *3*, 100056. https://doi.org/10.1016/j.crneur.2022.100056

Luders, E., Clark, K., Narr, K. L., & Toga, A. W. (2011). Enhanced brain connectivity in long-term meditation practitioners. *NeuroImage*, *57*(4), 1308–1316. https://doi.org/10.1016/j.neuroimage.2011.05.075

Malinowski, P. (2013). Neural mechanisms of attentional control in mindfulness meditation. *Frontiers in Neuroscience*, *7*. https://doi.org/10.3389/fnins.2013.00008

Nam, S., & Toneatto, T. (2016). The Influence of Attrition in Evaluating the Efficacy and Effectiveness of Mindfulness-Based Interventions. *International Journal of Mental Health and Addiction*, *14*(6), 969–981. https://doi.org/10.1007/s11469-016-9667-1

Ouyang, G., Hildebrandt, A., Schmitz, F., & Herrmann, C. S. (2020). Decomposing alpha and 1/f brain activities reveals their differential associations with cognitive processing speed. *NeuroImage*, *205*, 116304. https://doi.org/10.1016/j.neuroimage.2019.116304

Robiner, W. N. (2005). Enhancing adherence in clinical research. *Contemporary Clinical Trials*, *26*(1), 59–77. https://doi.org/10.1016/j.cct.2004.11.015

Rodriguez-Larios, J., Bracho Montes de Oca, E. A., & Alaerts, K. (2021). The EEG spectral properties of meditation and mind wandering differ between experienced meditators and novices. *NeuroImage*, *245*, 118669. https://doi.org/10.1016/j.neuroimage.2021.118669

Saggar, M., King, B., Zanesco, A., MacLean, K., Aichele, S., Jacobs, T., Bridwell, D., Shaver, P., Rosenberg, E., Sahdra, B., Ferrer, E., Tang, A., Mangun, G., Wallace, B. A., Miikkulainen, R., & Saron, C. (2012). Intensive training induces longitudinal changes in meditation state-related EEG oscillatory activity. *Frontiers in Human Neuroscience*, *6*. https://doi.org/10.3389/fnhum.2012.00256

Voytek, B., Kramer, M. A., Case, J., Lepage, K. Q., Tempesta, Z. R., Knight, R. T., & Gazzaley, A. (2015). Age-Related Changes in 1/f Neural Electrophysiological Noise. *Journal of Neuroscience*, *35*(38), 13257–13265. https://doi.org/10.1523/JNEUROSCI.2332-14.2015

Wong, W. P., Hassed, C., Chambers, R., & Coles, J. (2016). The Effects of Mindfulness on Persons with Mild Cognitive Impairment: Protocol for a Mixed-Methods Longitudinal Study. *Frontiers in Aging Neuroscience*, *8*. https://www.frontiersin.org/articles/10.3389/fnagi.2016.00156
