# Supplementary material for: Augmenting complex and dynamic performance through mindfulness-based cognitive training: An evaluation of training adherence, trait mindfulness, personality and resting-state EEG
Source: PLoS One. 2024 May 20;19(5):e0292501. doi: 10.1371/journal.pone.0292501 (PMC11104625; doi:10.1371/journal.pone.0292501)
Supplement: Attachment — Submitted filename: Second_Response_to_Reviewers.docx [file pone.0292501.s006.docx]

**PLOS ONE: PONE-D-23-29669**

**Response to Reviewers**

We thank the Reviewers for their final minor comments on our manuscript. Each comment has been carefully considered and amended in the manuscript to suit.

Please find enclosed the revised manuscript, which includes changes intended to fully address all remaining Editor and Reviewers’ comments.

All comments are shown in grey, while our responses are coded in black. All text snippets from the manuscript are *italicized,* with new content depicted in **bold**.

**Response to Editor**

1) Please review your reference list to ensure that it is complete and correct. If you have cited papers that have been retracted, please include the rationale for doing so in the manuscript text, or remove these references and replace them with relevant current references. Any changes to the reference list should be mentioned in the rebuttal letter that accompanies your revised manuscript. If you need to cite a retracted article, indicate the article’s retracted status in the References list and also include a citation and full reference for the retraction notice.

The reference list has been thoroughly reviewed to ensure correctness and completeness. All numbered references appearing in the reference list are evident in-text. One paper, Colzato et al. (2015) has been removed from the references (previously reference #94) due to retraction for fraudulent data. In place of this one experimental study example, we have provided a reference (#51) to review work by Cahn & Polich (2006) which describes multiple studies reporting changes in electrophysiology in novices. We thank the editor for bringing this to our attention.

**Response to Reviewer 2**

Thank you for thoroughly addressing my previous comments. The manuscript overall is much improved and considered acceptable for distribution. Only minor comments remain:

2) Wording in abstract sounds like enhancements were seen in resting state EEG when no changes were observed pre and post intervention. Suggest revision to make more clear.

We thank the reviewer for ensuring clarity within our abstract. The manuscript’s abstract has been amended to make it clear that changes were not observed pre and post intervention:

*Human performance applications of mindfulness-based training have demonstrated its utility in enhancing cognitive functioning. Previous studies have illustrated how these interventions can improve performance on traditional cognitive tests, however, little investigation has explored the extent to which mindfulness-based training can optimise performance in more dynamic and complex contexts. Further, from a neuroscientific perspective, the underlying mechanisms responsible for performance enhancements remain largely undescribed. With this in mind, the following study aimed to investigate how a short-term mindfulness intervention (one week) augments performance on a dynamic and complex task (target motion analyst task; TMA) in young, healthy adults (n = 40, age range = 18 - 38). Linear mixed effect modelling revealed that increased adherence to the web-based mindfulness-based training regime (ranging from 0-21 sessions) was associated with improved performance in the second testing session of the TMA task, controlling for baseline performance.* ***Analyses of resting-state electroencephalographic (EEG) metrics demonstrated no change across testing sessions. Investigation of*** *additional individual factors demonstrated* ***that*** *enhancements associated with training adherence remained relatively consistent across varying levels of participants’ resting-state EEG metrics, personality measures (i.e., trait mindfulness, neuroticism, conscientiousness), self-reported enjoyment and timing of intervention adherence. Our results thus indicate that mindfulness-based cognitive training leads to performance enhancements in distantly related tasks, irrespective of several individual differences. We also revealed nuances in the magnitude of cognitive enhancements contingent on the timing of adherence, regardless of total volume of training. Overall, our findings suggest that mindfulness-based training could be used in a myriad of settings to elicit transferable performance enhancements.*

3) Please remove language from correlational analyses that imply causation (e.g. sub header “effect of individual personality traits” – personality traits were not manipulated. Causational language is seen in other examples throughout text). I understand that the authors are referring to the main effects from each analysis, but within the discussion this language suggests the interpretation of the relationship as causal

We have amended discussion titles to remove any potential associations with causation (i.e., deleted “effect of”). In addition to this, we have reviewed word choices throughout the discussion to better demonstrate correlational relationships and associations within our data, not causal. Some examples are shown below:

Page 29: *In line with our hypotheses, some individual factors* ***were significantly associated*** ***with*** *the results, however, the most consistent predictor among models was adherence to the cognitive training regime.*

Page 30: *To further characterise the* ***neural correlates*** *of mindfulness-based cognitive training, we examined how the timing of adherence might influence its effects.*

Page 30: *Strikingly, our training regime* ***was correlated with*** *individual enhancements in performance even across a short time frame (up to 15 minutes per day, for one week)…*

Page 31: *In addition to timing of adherence, we investigated how individual-level enjoyment of the mindfulness exercises* ***was associated with*** *performance outcomes.*

Page 32: *Here, we found that lower levels of dispositional mindfulness were* ***correlated*** ***with*** *more distinct differences in lower and higher levels of adherence (similar to higher IAF, flatter slopes and higher 1/f intercepts; see below). That is, across the entire testing session, greater adherence* ***was related to*** *superior performance. For individuals with higher* ***self-reported*** *dispositional mindfulness,* ***the effect of adherence*** *was pronounced only towards the second half of the testing session, with more similar performance at the beginning of testing.*

Page 35: *On top of this, we found that individual neural characteristics at baseline* ***were not associated with*** *the general effects of mindfulness adherence.*

Page 35: *For example, higher levels of IAF, flatter slopes and higher 1/f intercepts demonstrated more* ***distinct relationships to*** *levels of high versus low adherence (i.e., more adherence was* ***correlated with*** *superior performance and less adherence* ***was correlated*** ***with*** *poorer performance across the entire testing session).*

Page 29: *The current study aimed to investigate how a one-week mindfulness-based cognitive training regime can augment performance in more dynamic tasks (i.e., the target motion analyst task [23]) while recognising the* ***influence*** *of individual differences.*
